# Interbrain cortical synchronization encodes multiple aspects of social interactions in monkey pairs

**DOI:** 10.1038/s41598-018-22679-x

**Published:** 2018-03-29

**Authors:** Po-He Tseng, Sankaranarayani Rajangam, Gary Lehew, Mikhail A. Lebedev, Miguel A. L. Nicolelis

**Affiliations:** 1 Department of Neurobiology, Duke University, Durham, NC 27710 USA; 2 Duke University Center for Neuroengineering, Duke University, Durham, NC 27710 USA; 3 Department of Biomedical Engineering, Duke University, Durham, NC 27708 USA; 4 Department of Psychology and Neuroscience, Duke University, Durham, NC 27708 USA; 5 Department of Neurology, Duke University, Durham, NC 27710 USA; 6 Edmund and Lily Safra International Institute of Neurosciences, Natal, 59066060 Brazil

## Abstract

While it is well known that the primate brain evolved to cope with complex social contingencies, the neurophysiological manifestation of social interactions in primates is not well understood. Here, concurrent wireless neuronal ensemble recordings from pairs of monkeys were conducted to measure interbrain cortical synchronization (ICS) during a whole-body navigation task that involved continuous social interaction of two monkeys. One monkey, the passenger, was carried in a robotic wheelchair to a food dispenser, while a second monkey, the observer, remained stationary, watching the passenger. The two monkeys alternated the passenger and the observer roles. Concurrent neuronal ensemble recordings from the monkeys’ motor cortex and the premotor dorsal area revealed episodic occurrence of ICS with probability that depended on the wheelchair kinematics, the passenger-observer distance, and the passenger-food distance – the social-interaction factors previously described in behavioral studies. These results suggest that ICS represents specific aspects of primate social interactions.

## Introduction

Observing the behaviors of others is essential for primates, including humans, to be able to handle the complex dynamics of their social groups. Such observations may allow individuals to learn social ranks, recognize threats and potential allies, as well as learn new motor skills^1,2^. Experiments in monkeys have demonstrated that, while an animal observes the actions performed by a different subject, frontal and parietal neurons of the observer respond as if the observer performed the same action by itself^3–14^. Such cortical neurons, which represent the actions of others, are classically known as “mirror neurons”^15^.

Studies of mirror neurons have provided insights on how cortical neuronal ensembles mediate social interactions through imitation and motor cooperation^16–22^. Several of these studies employed interactive tasks where monkey pairs cooperated or competed^14,23,24^. Such experiments usually employed single-unit recordings from one of the subjects’ brains, but not large-scale neuronal ensemble recordings obtained simultaneously from both brains of the interacting animals. This shortcoming resulted mostly from the major technical challenges involved in obtaining concurrent neuronal recordings from multiple subjects. Because of this limitation, the neuronal correlates of social interaction among multiple subjects have not been fully investigated in studies dealing with mirror neuron activity.

In the present study, we overcame this technical barrier by introducing a new paradigm that allowed multichannel wireless recording from the brains of a monkey pair engaged in a whole-body navigation task. Using this new neurophysiological approach, we asked how cortical activity recorded simultaneously from two monkeys could reflect such parameters as the animals’ relative position in space and their closeness to food – crucial factors determining primate social interaction^25–28^. In the whole-body navigation paradigm, pairs of monkeys alternatively played one of two distinct roles from day to day: while one monkey, the passenger, was carried by a motorized wheelchair, another, the observer, was seated in a stationary chair, watching the passenger’s whole-body movement. Both monkeys were motivated to attend to the wheelchair movements because they were both rewarded upon the passenger reaching the target: the passenger collected grapes, while the observer received juice. Our analysis showed that ICS between the two monkeys reflected the passenger’s whole-body movements and spatial location, the distance between the passenger and observer, and the distance between the passenger and food reward.

## Results

**Figure 1:**
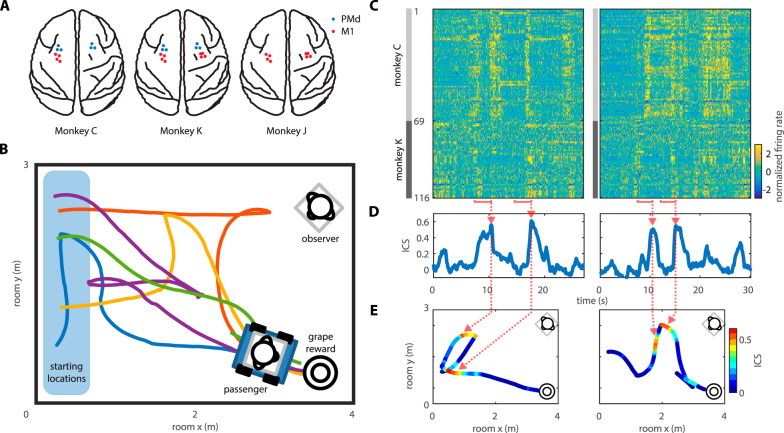
Interbrain cortical synchronization (ICS) during the navigation task. (**A**) Locations of cortical implants in three monkeys (**C**,**J**, and **K**). Neuronal-ensemble recordings were conducted in M1 (red dots) and PMd (blue dots), in both hemispheres. (**B**) The experimental setup. Two monkeys (passenger and observer) were placed in a 5.0-by-3.9 m room. The passenger sat in an electrically actuated wheelchair. The observer sat in a stationary chair placed in the corner of the room. During each trial, the passenger moved from a starting location (shown on the left) to a stationary grape dispenser. Five representative routes of the wheelchair are plotted in different colors. These routes were randomly generated by a computer program. (**C**) Color plots of neuronal-ensemble activity for two representative trials. Each horizontal line corresponds to a unit. Color represents normalized (z-scored) firing rate of 69 units were recorded in monkey C (observer in this experiment) and 47 in monkey K (passenger). Episodes of ICS are marked by red horizontal lines. (**D**) Continuous evaluation of ICS for the trials shown in (**C**). Instantaneous values of the distance correlation were computed with a sliding window, of the same 3-s width as the red bars in (**C**). Correlation peaks are marked by arrows. (**E**) Wheelchair routes for the same trials as in (**C**) and (**D**). The routes are color-coded to indicate ICS.

Experiments were conducted in three female monkeys, monkeys C, J and K. Of these, monkey C was the most dominant, as judged by the feeding priority in conflict over food^29–31^, followed by monkey K, and then by monkey J (see Methods: Social ranking). Monkeys were chronically implanted with multiple cortical multielectrode arrays. A monkey pair participated in each experimental session; monkey pairs C-K and C-J were tested. Neuronal ensemble activity was recorded in both monkeys simultaneously, using a 256-channel wireless recording system^32,33^. In monkey J, we recorded bilaterally from the primary motor cortex (M1), and in monkeys C and K, bilaterally from M1 and dorsal premotor cortex (PMd) (Fig. 1A). The number of recorded units ranged, depending on the session, 66–68 in monkey J, 70–90 in monkey C (M1, 43–54; PMd, 27–36), and 43–48 in monkey K (M1, 30–33; PMd, 43–48).

During the experiments, animals were seated in their monkey chairs and placed inside a 5.0m-by-3.9 m room (Fig. 1B). The chair of the monkey, called observer, remained stationary. The chair of the other monkey, called passenger, was mounted on an electrically actuated cart that traveled freely in the room. The task consisted of the passenger navigating toward a grape dispenser to collect a grape. At the time the passenger obtained the grape, the observer also received a juice reward. The grape dispenser was situated in the corner of the room; the observer stayed in another corner. The passenger started navigation from a location near the wall opposing the grape dispenser and observer. For both monkey pairs (C-K and C-J), the passenger and observer roles were alternated among monkeys in different experiments. The wheelchair was moved by a computer program along a randomly generated trajectory.

### Interbrain cortical synchronization between the passenger and observer

We observed that cortical activity concurrently recorded in both the passenger and observer exhibited episodic ICS, where the correlation of neuronal firing patterns between the two monkeys was significantly higher than the one obtained for permuted data (i.e., null distribution). While the cause of this ICS was not apparent in individual trials, statistical analysis of the ICS episodes (see Methods: Episodic ICS) showed that their probability of occurrence depended on wheelchair position and velocity, the distances between the passenger and grape dispensers, and the distance between the passenger and observer. Figure 1C–E shows two representative trials containing several ICS episodes, with correlation coefficients as high as 0.5. ICS episodes constituted 19.7% ± 2.1% (mean ± standard error) of the total session time for monkey pair C-K, and 35.7% ± 4.7% for C-J (Supplementary Fig. S1).

To determine the factors influencing such an ICS, we calculated the probability of ICS episodes as a function of different parameters of the wheelchair movements: translational and rotational velocity, room coordinates of the wheelchair, distance from the wheelchair to the grape dispenser, and distance from the wheelchair to the observer (see Methods: ICS probability). We found that ICS probability depended on each of these parameters, and was also influenced by monkey pair composition and task roles (passenger vs. observer) assigned to each monkey.

**Figure 2:**
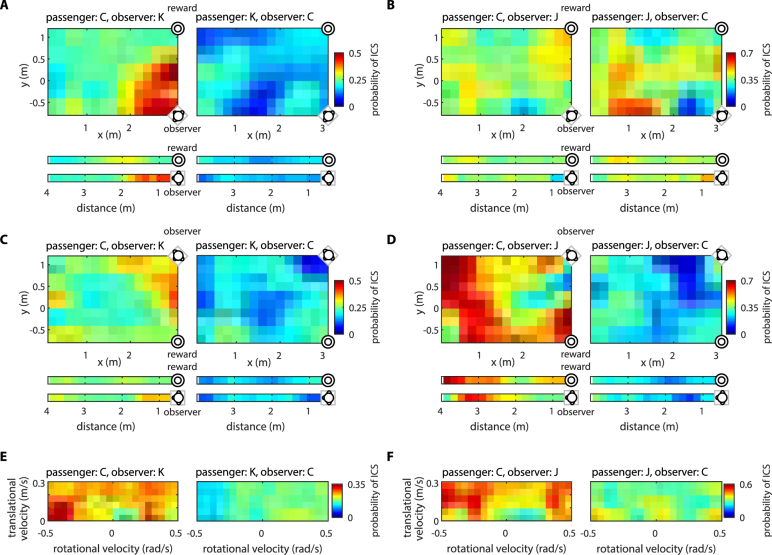
The dependence of ICS on wheelchair position and velocity, expressed as conditional probability of ICS episodes. (**A**–**D**) Probability of ICS episodes as a function of room x,y coordinates. The values of probability at each pixel are averaged with immediate neighbors and are color coded. The one-dimensional bars below each two-dimensional plot show the dependence of probability on the distance between the passenger and food reward (top), and between the passenger and the observer (bottom). The results for monkey pair C-K are shown in (**A,C**) and for monkey pair C–J in (**B**,**D**). Monkey C was either passenger (panels on the left) or observer (panels on the right). In (**A**,**B**) the observer was in the right corner of the room (relative to the passenger at the starting location), and the grape dispenser was in the left corner. In (**C,D**), the locations of the observer and grape dispenser were swapped. (**E,F**) Color-coded probability of ICS episodes as a function of wheelchair velocity for monkey pairs C–K (**E**) and C–J (**F**). Horizontal axis corresponds to rotational velocity, and vertical axis corresponds to translational velocity. (See Fig. S4 for the number of samples contributed to each pixel).

Figure 2 illustrates how ICS probability depended on the wheelchair position (Fig. 2A–D) and velocity (Fig. 2E,F). When monkey C was paired with monkey K, and C was the passenger, episodes of high ICS became more frequent when the distance between the monkeys decreased (Fig. 2A,C, left panels, where the two distributions look like mirrored images as the observer and the reward swapped locations). This relationship reversed when monkey C was the observer (Fig. 2A,C, right panels): synchrony episodes occurred less frequently when the monkeys were close to each other. For the same monkey pair, the probability of ICS episodes also depended on the wheelchair velocity. This dependence also reversed after the monkeys’ roles changed (compare left and right panels in Fig. 2E). Similar effects were observed for monkey pair C-J. Wheelchair position, velocity and monkey roles influenced the probability of ICS (Fig. 2B,D,F). Notably for this monkey pair, higher levels of ICS were frequent when monkey C, as the passenger, was far away from monkey J (Fig. 2D, left), the observer. When C approached J, ICS episodes became less frequent. The dependence of ICS on the passenger’s position changed dramatically after C became the observer (Fig. 2D, right), as did the dependence of ICS on wheelchair velocity (Fig. 2F).

In addition to the effect of the distance between the monkeys, ICS probability was affected by the distance between the passenger and the grape dispenser (Fig. 2A–D). These ICS patterns depended on the monkey pair composition. For example, in monkey pair C-K, the probability of ICS episodes increased when the passenger (C or K) approached the grape dispenser (Fig. 2A,C; Spearman correlation = −0.28 ± 0.18). Conversely, for pair C-J, the ICS decreased when the passenger (C or J) was close to the grape dispenser (Fig. 2B,D; Spearman correlation = 0.47 ± 0.17).

While ICS probability clearly depended on where the passenger was positioned relative to the observer and the grape dispenser, these neural patterns could also reflect room landmarks, for example, left versus right corner. We tested for this possibility by swapping the locations of the grape dispenser and observer on different sessions. This manipulation produced a reversal of the ICS patterns (e.g., compare Fig. 2A and C), indicating that room landmarks were not as important as the positions of the observer and the grape dispenser.

ICS probability also depended on the wheelchair velocity. In the example of Fig. 2E, the dominant monkey C was the passenger and monkey K was the observer. In these settings, ICS probability increased when the wheelchair had high rotational velocity. However, when the monkeys’ roles were swapped, the ICS probability did not vary with increases of the wheelchair rotational velocity. ICS patterns depended on the wheelchair velocity in the monkey pair C-J, as well (Fig. 2F).

Since in the monkey pair C-K we recorded from M1 and PMd in both animals, we could compare the engagement of each of these cortical areas in ICS. For every ICS episode, determined by the analysis for the entire neuronal sample, we measured whether a particular pair of cortical areas exhibited ICS (permutation test, p < 0.05). When monkey K was the observer, M1-to-M1 synchronization was found to be most frequent (60.5% ± 1.3% of the episodes), followed by M1-to-PMd (51.5% ± 1.0%) and PMd-to-PMd (42.5% ± 1.4%). The same trend was found when monkey C was the observer: 53.0% ± 1.3% for M1-to-M1, 47.0% ± 1.0% for M1-to-PMd, and 43.6% ± 1.4% for PMd-to-PMd.

### Neuronal modulation to wheelchair velocity and acceleration

**Figure 3:**
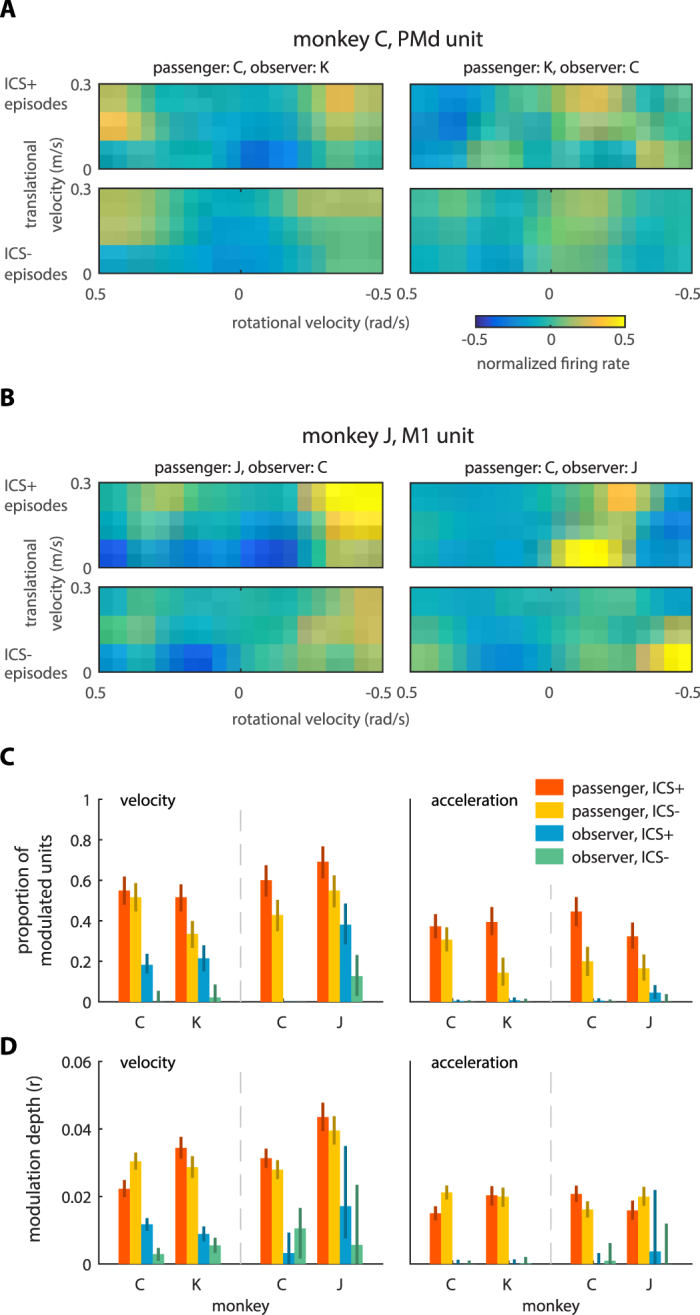
Modulation of passenger’s and observer’s units to wheelchair kinematics. (**A**) Modulation patterns to rotational (horizontal axis) and translational (vertical axis) velocity in a representative PMd unit recorded in monkey C. Color represents normalized (z-scored) firing rate. In these experiments, monkey C was paired with monkey K, and acted as passenger (left panels) or observer (right panels). ICS episodes were detected, and neuronal modulation was assessed separately when these episodes were present (ICS+, top panels) and absent (ICS−, bottom panels). (**B**) Modulation patterns in an M1 unit recorded in monkey J. Monkey J was paired with monkey C. Conventions as in (**B**). (**C**) Bar plots representing average proportion of units modulated to the wheelchair velocity (left panel) and acceleration (right panel) for different monkey pairs, monkeys, and monkey roles (passenger or observer). Values are shown separately for the presence and absence of ICS episodes. Error bars represent 95% confidence interval obtained by 1,000 bootstrap replicates. (**D**) Averaged neural modulation depth to the wheelchair velocity (left panel) and acceleration (right panel). Conventions as in (**C**).

Having established that ICS depended on the passenger’s position and velocity, we examined the relationship between the presence of ICS and the modulation of neuronal firing rates to position and velocity, in both the passenger’s and observer’s cortex. For that, we first evaluated neuronal modulation patterns to wheelchair kinematics, i.e. velocity and acceleration (Fig. 3). This analysis revealed that the passenger had more velocity and acceleration modulated units in both M1 and PMd (χ^2^(1) > 9.35, p < 0.05). In the passenger’s PMd, 40.6% and 19.5% units (data from all monkey pairs combined) were modulated to velocity and acceleration, respectively, whereas in the observer’s PMd these values dropped to 0.8% and 0%. Considering M1, the corresponding values were 43.8% and 15.5% for the passenger and 0.5% and 0% for the observer. The dependence of neuronal firing rate on different parameters of navigation was quantified using modulation depth as a metric (see Methods: Modulation depth). Modulation depth for both velocity and acceleration was on average higher in the passenger compared to the observer (ANOVA, F(1,1434) > 270.2 for monkey role, p < 0.05; See Table S1, S2 and Methods: ANOVA for modulation depth). Additionally, significant differences were found when M1 modulation depth was compared to that of PMd (permutation test, p < 0.05). Monkey pair-dependent effects were found: in monkey C, modulation was stronger in PMd than M1 when C was paired with K but weaker when C was paired with J (permutation test, p < 0.05) (Table S9).

To assess whether there was any correspondence between the modulation depth values during navigating and observing, we calculated the correlation coefficient between the neuronal population features recorded in different areas (Fig. S2). We found significant correlations for both M1 (Spearman correlation rho = 0.24–0.64, p < 0.05) and PMd (rho = 0.48–0.81, p < 0.05).

The presence or absence of ICS clearly correlated with changes in modulation of neuronal rates to wheelchair kinematics (Fig. 3A,B, top vs. bottom). Figure 3A,B shows two representative units. The first was recorded in monkey C’s PMd when C was paired with K (Fig. 3A). When monkey C was the passenger, this PMd unit was non-directionally modulated to rotational velocity: its firing rate increased for both rightward and leftward rotations (see X-axis). Yet, during ICS episodes in which monkey C became the observer, this PMd unit’s velocity modulation pattern became more prominent since its neuronal firing rate increased for rightward rotations and decreased for leftward rotations (Fig. 3A, right, see X-axis). The second example illustrated is an M1 unit recorded in monkey J when it was paired with monkey C (Fig. 3B). This M1 unit responded to both translational and rotational velocity and its firing increased during the ICS episodes. As before, this M1 unit also changed its velocity modulation properties when the monkeys’ roles were reassigned. When monkey J was the passenger, the unit increased firing rate in response to wheelchair rightward rotation combined with forward movement. When monkey J was the observer, the unit decreased firing rate to this pattern of wheelchair movement.

For the entire neuronal sample, more units were modulated to the wheelchair velocity in the presence of synchrony episodes (39.0% ± 5.0%, data from all monkeys combined) than in their absence (23.9% ± 3.2%) (Fig. 3C, left). This difference was statistically significant for all monkey combinations (χ^2^(1) = 70.1, p < 0.05), and was not related to changes in absolute firing rate between the presence and absence of ICS (see Supplementary Materials: Controlling firing rate in ICS). The analysis of population-averaged modulation depth also showed a clear main effect of the presence of synchronization (ANOVA, F(1,1434) = 7.6, p < 0.05) (Table S1; Fig. 3D, left). Thus, we observed both a significantly larger number of modulated units and an increase in modulation depth in the individual units during the ICS episodes. Additionally, ANOVA showed the main effect of monkey dominance rank. In this analysis, monkey C had a higher dominance rank than the others. We found weaker neuronal modulations in the more dominant monkeys (F(1,1434) = 13.6, p < 0.05), and the modulation depth also depended on the monkey pairs (C-K or C-J pair, F(1,1434) = 9.3, p < 0.05). The analysis of neuronal modulations to acceleration showed that more units were modulated to acceleration in the presence of ICS episodes (19.3% ± 1.2%; data from all monkeys combined) than in their absence (10.3% ± 1.3%) (χ^2^(1) = 38.8, p < 0.05) (Fig. 3C, right). However, population-average modulation depth to wheelchair acceleration did not increase during the synchronous episodes (ANOVA, F(1,1434) = 0.03, p = 0.86), and did not depend on the dominance rank (F(1,1434) = 0.06, p = 0.81) or monkey pair (F(1,1434) = 0.39, p = 0.53) (Table S2; Fig. 3D, right).

### Spatial modulation

**Figure 4:**
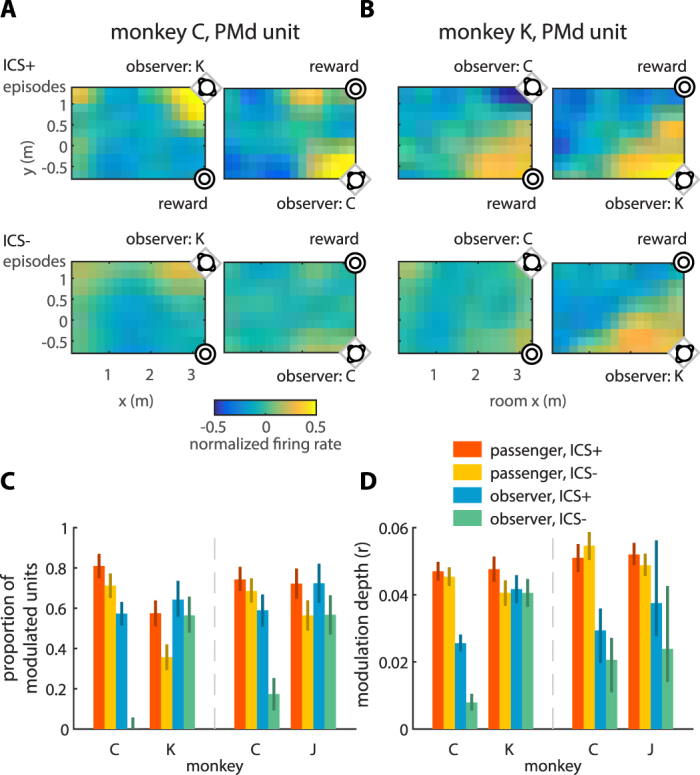
Modulation of passenger’s and observer’s units to the wheelchair room coordinates. (**A**) Modulation of a PMd unit recorded in monkey C. Color represents normalized (z-scored) firing rate. In these experiments, monkey C was paired with monkey K, and acted as passenger (left panels) or observer (right panels). The observer was seated in the left corner (left panels) or right corner (right panels) (relative to the passenger at the starting location). Neuronal modulation was assessed separately when ICS episodes were present (ICS+, top panels) or absent (ICS−, bottom panels). (**B**) Modulation of a PMd unit recorded in monkey K. Monkey K was paired with monkey C. Conventions as in (**A**). (**C**) Bar plots representing average proportion of units modulated to wheelchair position for different monkey pairs, monkeys, and monkey roles (passenger or observer). Proportions are shown separately for the presence and absence of ICS episodes. Error bars represent 95% confidence interval obtained by 1,000 bootstrap replicates. (**D**) Averaged modulation depth. Conventions as in (**C**).

M1 and PMd units were modulated to wheelchair room position in both the passenger’s and observer’s brains (Fig. 4). The color plots of Fig. 4A,B show two representative PMd units whose firing rate changed when the passenger traveled to different room locations. These spatial modulation patterns were affected by the presence or absence of ICS episodes and monkey roles. Figure 4A shows a PMd unit recorded in monkey C when it was paired with monkey K. The neuronal rate increased with decreasing distance between the monkeys when monkey C was the passenger or observer. In both cases, the unit’s firing response to the distance between the monkeys increased during the ICS episodes (Fig. 4A, top vs bottom). The second illustrated unit was recorded in monkey K’s PMd. This unit’s rate decreased when monkey K was the passenger and approached monkey C (Fig. 4B, left). Conversely, the firing rate increased when monkey K approached the grape dispenser. The response pattern of the same unit was very different when monkey K was the observer (Fig. 4B, right). In this case, the neuronal rate increased when monkey K approached monkey C, but decreased when it approached the grape dispenser. For both the passenger and observer roles of monkey K, spatial modulation was stronger during the synchrony episodes (Fig. 4B, top vs bottom).

Several analyses were conducted to assess different features of spatially-dependent modulations of neuronal rates. We first assessed neuronal rate as a function of room x,y coordinates. Next, we analyzed the dependence of firing rate on the distance between the monkeys. Lastly, we analyzed the role of the distance from the passenger to grape dispenser. In each analysis, we used the same ANOVA as in the previous section (see Methods: ANOVA for modulation depth).

The analysis of modulation to x,y room coordinates showed that more units (χ^2^(1) = 153.2, p < 0.05) were modulated to wheelchair position in the passenger (55.5% ± 6.9%, data from all monkeys combined) compared to the observer (30.9% ± 14.1%) (Fig. 4C; Table S9). Consistent with this result, average spatial modulation depth was higher in the passenger than observer in all cases (ANOVA, F(1,1434) = 88.1, p < 0.05; Methods: ANOVA for modulation depth; Table S3). The presence of ICS episodes increased spatial modulation strength (F(1,1434) = 31.8, p < 0.05). When expressed as the percentage of spatially modulated units, this effect was significant for all monkey pairs (chi χ^2^(1) = 179.0, p < 0.05): 66.7% ± 2.5% of units (all monkeys combined) were significantly modulated to wheelchair location in the presence of ICS episodes and 43.8% ± 5.2% in their absence. Additionally, modulation depth was lower for more dominant monkeys (F(1,1434) = 8.6, p < 0.05) (Fig. 4D; Table S3).

**Figure 5:**
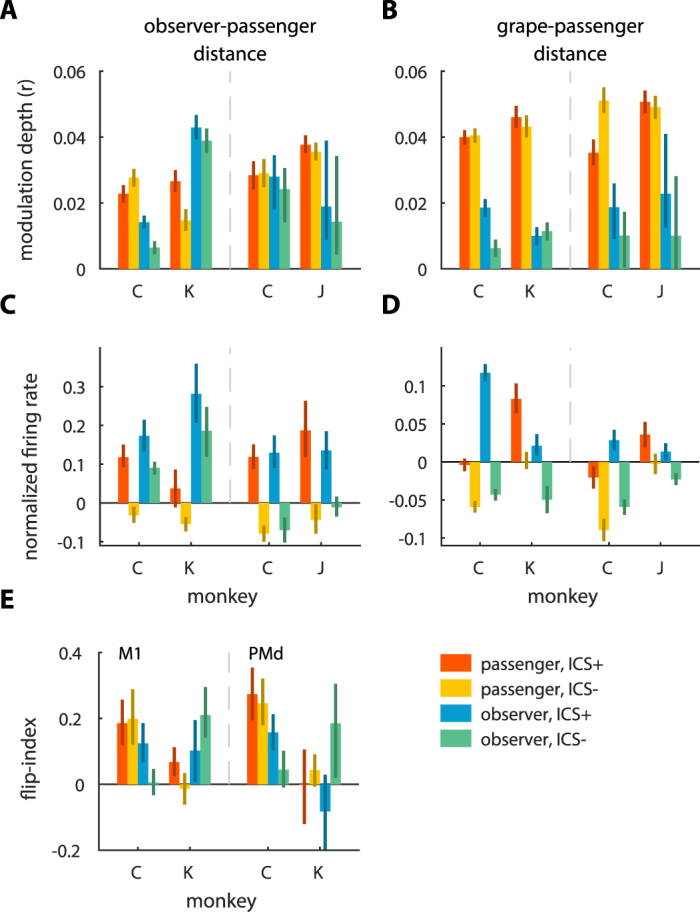
Neuronal activity patterns across monkeys and ICS conditions. (**A**) Bar plots represent average modulation depth to the distance between the passenger and observer. Results are presented separately for different monkey pairs, monkeys, monkey roles (passenger or observer), and the presence/absence of ICS episodes. Error bars represent 95% confidence interval obtained by 1,000 bootstrap replicates. Panels (**B–E**) use the same conventions as in (**A**). (**B**) Average modulation depth to the distance between the passenger and grape dispenser. (**C**) Average normalized firing rates for the close (<1 m) distance between the passenger and observer. (**D**) Average normalized firing rates for the close (<1 m) distance between the passenger and grape dispenser. (**E**) Average flip-index for M1 (left) and PMd units (right).

In terms of the responses to the distance between the monkeys, 47.6% ± 4.3% of units (data from all monkeys combined) were modulated in the passenger and 29.5% ± 4.1% in the observer. The difference between these proportions was statistically significant (χ^2^(1) = 102.0, p < 0.05). Modulation strength in the passenger was stronger than in the observer (ANOVA, F(1,1434) = 7.2, p < 0.05; Methods: ANOVA for modulation strength) (Table S4). The presence of ICS resulted in stronger modulation (F(1,1434) = 11.6, p < 0.05) (Fig. 5A). In addition, another two ANOVA factors, social rank and role, also showed significant main effects: in a monkey pair, stronger modulations occurred in the monkey with a higher dominance rank (F(1,1434) = 12.1, p < 0.05), and in the monkey that acted as observer (F(1,1434) = 7.2, p < 0.05) (Table S4). Since it has been shown that mirror neurons are modulated differently to actions occurring in the extrapersonal and peripersonal space^4^, we conducted a separate examination of neuronal activity recorded when the inter-monkey distance was less than 1 m. For this distance range, neuronal rates significantly increased during the ICS episodes in both the passenger and observer (ANOVA, F = (1,1434) = 358.7, p < 0.05; see Methods: ANOVA for firing rates) (Fig. 5C; Table S5). The rates were lower for the dominant monkey in the pair (F = (1,1434) = 7.2, p < 0.05), and units in the observer had higher rates compared to the passenger (F = (1,1434) = 43.6, p < 0.05).

Modulation to distance between the passenger and the grape dispenser was found in 52.0% ± 5.4% of the passenger’s units and in a small proportion (5.3% ± 3.4%, χ^2^(1) = 418.0, p < 0.05) of the observer’s units. Individual units were more strongly modulated in the passenger than the observer (ANOVA, F(1,1434) = 270.6, p < 0.05; Methods: ANOVA for modulation strength) (Fig. 5B; Table S6). Additionally, modulation strength increased during ICS episodes (F(1,1434) = 5.8, p < 0.05) (Fig. 5B; Table S6). For the 1-meter zone around the grape dispenser, neuronal rates were higher during the ICS episodes (ANOVA, F(1,1434) = 610.9, p < 0.05; see Methods: ANOVA for firing rates) and when the monkey was the subordinate monkey (F(1,1434) = 54.3, p < 0.05) or the observer monkey (F(1,1434) = 4.5, p < 0.05) (Fig. 5D; Table S7).

Finally, we analyzed the changes in neuronal spatial modulation after the locations of the observer and grape dispenser were swapped. We tested whether the swapping symmetrically reflected the spatial modulation pattern relative to the longitudinal axis of the room. Our analysis showed that a symmetric reflection of the spatial modulation patterns occurred only for monkey pair C-K (average flip-index = 0.176 ± 0.018, bootstrapping test, p < 0.05; see Methods: flip-index), but not C-J (flip-index = −0.039 ± 0.015, bootstrapping test, p < 0.05). The reflection was clearer for units recorded in monkey C, the most dominant monkey, (flip-index of 0.22 ± 0.03) than monkey K (flip-index of 0.11 ± 0.03) (bootstrapping test, p < 0.05) (Fig. 5E). The ANOVA for the flip index did not show differences in units’ flip-index for cortical area, monkey role, nor the presence of ICS (ANOVA, F(1,218) < 1.7, p > 0.19; see Methods: ANOVA for flip-index; Table S8). In monkey pair C-J, the pattern change could not be described as a symmetric reflection (flip-index = −0.039 ± 0.015, bootstrapping test, p < 0.05), suggesting that cortical neuronal modulation was affected by the arrangement of the observer and dispenser positions in room-centered coordinates.

## Discussion

In this study, a whole-body navigation/observation task, combined with simultaneous wireless recordings of cortical neuronal ensemble activity from pairs of monkeys, was employed to investigate the neuronal correlates of spatial social interactions in primates. One monkey (the observer) remained stationary while another monkey (the passenger) navigated using a robotic wheelchair. This paradigm allowed us to examine how the social interaction between the monkey pair was affected by whole-body movements of either the dominant or the subordinate animals. Simultaneous recordings of cortical ensemble activity from both the passenger and observer revealed that their M1 and PMd units modulated their firing rate in accordance to the roles of the monkey (passenger or observer), as well as the wheelchair position and velocity. Moreover, we found that cortical units located in the two monkey brains exhibited episodes of transient synchronized firing. The probability and magnitude of such ICS depended on the wheelchair kinematics, the distance between the monkeys, and the distance between the passenger and the food reward. Based on these findings, we propose that high ICS defines a fundamental neurophysiological manifestation underlying social interactions in primates, and likely, other animals.

Using concurrent wireless interbrain recordings, we identified the occurrence of episodes of social interaction even without considering behavioral measurements of such an interaction. In our approach, ICS was analyzed as a stochastic process, where the occurrence of synchronous episodes was described by conditional probability that depended on multiple parameters, including wheelchair position and velocity, inter-subject distance, and the distance between the passenger and food reward. The color maps of the conditional probability of such ICS episodes were similar to classical neuronal tuning curves, with the key difference that our metric represented the combined activity of multiple brains rather than the activity of an individual brain. Such ICS maps allowed us to assess the role of experimentally controlled parameters in social interaction. Evidently, it is possible – and likely - that other uncontrolled factors contributed to the ICS observed here. The contribution from factors like eye/head movements, lip smacking, facial expressions, eye contact, other whole-body signals^34–37^, and auditory responses (e.g., caused by subtle noise from the wheelchair motor) will have to be investigated in greater detail in future studies. These uncontrolled factors would only have a significant effect on neural activities if both monkeys attended to them simultaneously, and joint attention has been proposed as a basic mechanism for social interaction^38^.

Mathematically, ICS can be described as an occurrence of specific neuronal firing patterns, like zero-lag synchrony^39^, in a high-dimensional neuronal space composed of the activity of multiple brains. Here, we only considered neuronal firing patterns generated jointly by two monkey brains, but in the future, the same analytical approach employed in the present study could be expanded to characterize meaningful and effective social interactions, from a systems neurophysiological point of view, that occur in large groups of primates, and likely, in other animal species, like rats and mice. Concurrent EEG and brain imaging can also be employed to study the same type of ICS in human subjects. In fact, several recent publications employed functional magnetic resonance imaging (fMRI) to assess brain processing in human subjects engaged in interactive and social behaviors^40–52^. The term “hyperscanning” was coined for such fMRI studies^43^. In these studies, participants interacted simultaneously or sequentially through interfaces, such as videos^43–48,51^ and/or audios^49,52^. For example, one fMRI study^49^ found a high level of synchronization between the subjects that viewed the same segment of a popular movie. Voxel-to-voxel synchronization was observed in the visual and auditory cortical areas, including primary, secondary and association areas. Another study investigated neural correlates of verbal communications^49^. fMRI recordings were first conducted in a speaker and then the audio was replayed while fMRI was obtained from a listener. Correlation analysis of the obtained brain scans suggested that successful verbal communication requires brain-to-brain coupling. Such coupling occurred when the speaker and listener shared the same language, and it diminished when the listener was told a story in a foreign language. Similar brain-to-brain coupling was demonstrated for non-verbal communications in humans, including communication with gestures^47^ and facial expressions^44^. Interestingly, examination of the activity of each communicating brain alone failed to elucidate the extent of the neural circuitry involved in these interbrain interactions^46^. Recently, a dyadic imaging approach was developed^53,54^, where two participants laying side-by-side were scanned simultaneously in the same MRI scanner. Such a setting allowed face-to-face social interaction to be studied. In one study^50^, brain activity obtained while two subjects placed in the same scanner established eye contact was compared with fMRI data obtained while a subject visualized pictures of opened/closed eyes of another person. This comparison revealed that establishing eye contact with another person generates a very different pattern of brain activation than the one obtained when subjects just looked at pictures of human eyes. At this point, it is important to highlight that our study, as far as we can tell, is the first to employ concurrent wireless neuronal ensemble recordings in monkey dyads to demonstrate that ICS of cortical motor areas can be involved in representing multiple aspects of primate social interactions.

Additional knowledge on brain-to-brain coupling has been provided by brain-machine interface (BMI) studies where neural activity was simultaneously decoded from the brains of a group of subjects trained to control various external devices jointly. The term “Brainet” was recently coined by our laboratory to describe BMI implementations of this kind^55^. Historically, such multi-brain systems were first implemented in the 1970s using electroencephalographic (EEG) recordings in humans^41,56^. These early applications enabled a variety of cooperative tasks, including EEG-controlled music performance by multiple subjects^56,57^ and drawing of Lissajous curves by two participants synchronizing their alpha EEG rhythms^58^. In our lab, intracranial recordings from ensembles of monkey cortical neurons were utilized for a similar purpose: pairs or trios of monkeys learned to cooperatively control the reaching movements performed by a virtual arm using their combined cortical ensemble activity^55^.

Several factors contributed to the occurrence of ICS observed in our study. First, wheelchair movements triggered episodes of ICS because they simultaneously engaged cortical processing in both monkeys. When the wheelchair moved, the passenger’s M1 and PMd units responded to the animal’s whole-body displacement. Conversely, in the observer, equivalent cortical neuronal populations responded as a way to represent the passenger’s movements in a mirror fashion. In addition, the distance between the monkeys was an important social-interaction factor that affected the probability of the two monkey motor cortices being synchronized.

Furthermore, cortical activity in both monkeys’ brains was related to the expectation of simultaneously acquiring rewards, when the passenger monkey reached the region where it could collect grapes and the observer received a fruit juice reward. As such, this reward expectation defined another important way in which the monkey pairs interacted socially. Consequently, the distance between the passenger and reward was found to be correlated with the probability of ICS episodes. Although previous studies have considered behavioral manifestations of similar social-interaction factors, including joint displacement and relative position of group members^59,60^ and food seeking and consumption^29,61,62^, our study is the first to document the occurrence of ICS in at least two cortical motor areas, M1 and PMD, as the potential neurophysiological manifestations underlying such social interactions. In this context, our present finding extends previous results obtained in individual monkeys that showed that M1 and PMd neuronal ensembles also encode reward magnitude and expectation^63–65^. Here we showed that potential rewards are also encoded by ICS. Since responses to reward in the acting monkey likely represented dopaminergic inputs to M1 and PMd^64^, it is reasonable to suggest that similar dopaminergic effects could be present in the observer’s brain in our task. Taking this argument further, one could raise the hypothesis that dopaminergic effects during the observation of rewarded actions may explain the phenomenon of learning by observation^66,67^. In the context of the present study, ICS could be interpreted as a potential neuronal manifestation of social learning and even a mechanism to facilitate knowledge transfer from one subject to the other.

Interestingly, ICS also reflected high-order variables involved in social interactions, such as the composition of monkey pairs and their assigned roles in the task. Indeed, the magnitude of the ICS in a monkey pair changed when a passenger and an observer flipped their roles. For example, strong ICS was observed when monkey C and monkey K were close to each other and C was the passenger. Conversely, this ICS decreased when monkey C became the observer. These results could be related to the social hierarchy of the monkeys in our colony. In support of this suggestion, it was observed in previous studies that dominant monkeys freely roam in their surrounding space, while submissive monkeys suppress their behaviors in that space to avoid conflict^7,62,68,69^. In our experiment, monkey C’s dominance rank was higher than monkey K and J. This difference in the monkey’s social rank could translate into the effects we observed here. Irrespective of the social ranking of our monkey colony, it is reasonable to suggest that the one-meter space around the observer could be considered as “the zone of potential conflict” for the monkey pairs. This could explain the occurrence of prominent ICS when the passenger entered this zone.

Although ICS obviously was not related to direct connectivity between the monkeys’ brains, it is possible that the Hebbian rule “fire together, wire together” is a valid metaphor for describing the plastic neurophysiological changes resulting from continuous social interaction. Using this analogy, the enhancement of “social connectivity” between multiple animal brains, instead of synaptic connectivity, may be strengthened or weakened, through changes in ICS resulting from social contact. For example, if the passenger’s whole-body movement, associated with bursts of activity in the passenger’s M1 and PMd neurons, is consistently coupled with modulations in firing rate of the observer’s equivalent neurons, the two monkey brains get “effectively synchronized”, although they are not interconnected directly. In real-life situations, such social connectivity is likely to result in causal relationships. For example, cortical activity of a monkey engaged in a behavior would translate into the cortical activity of a nearby monkey, and so on, until the repetition of this social interaction could effectively reshape the interbrain functional connectivity of an entire social group. Our lab has called the resulting chain of synchronized brains a Brainet^55^. According to our view, the occurrence of strong episodes of ICS would be the major determinant leading to the creation of such Brainets through animal social interactions occurring in their natural environments. In this context, such Brainets could include a large number of individual brains.

Given this new view on how social interactions are represented by the combined activity of multiple brains and how ICS could underlie social learning, it would be of interest to compare our findings with the previous theoretical framework built around the notion of mirror neurons. Several observations in our study are reminiscent of previous findings related to mirror neuron activity. For example, the observation that a significant fraction of M1 and PMd units in the observer’s brain are modulated to the wheelchair’s rotation and translational velocity is consistent with the literature on mirror neurons representing observation of actions in premotor^4,8,15,16^ and motor^6,12^ cortical areas. Mirror neurons are described as cortical neurons that respond the same way when a subject performs or observes an action. This definition is only marginally applicable to our findings because, generally, a given PMd or M1 neuron had different tuning patterns during the monkey’s navigating or observing. Yet, the modulation depth for navigation was correlated with the modulation depth for observation, suggesting the existence of typical mirror neuron activity. Additionally, average modulation depth was higher in the passenger than the observer. While it is tempting to attribute this result to a stronger representation of self-motion in M1 and PMd, as opposed to motion observation, it is also possible that the observer was not attentive enough to the passenger’s movements in our experiments. Indeed, paying attention to the passenger was not required by the task. It would be of interest in the future to employ a behavioral task where the observer must attend to the passenger to obtain the reward. In one previous study^70^, monkeys were required to attend to the movements performed by a robot, and a significant portion of PMd neurons (~20%) became modulated to spatial attention orientation.

The other result that somewhat resembles previous findings on mirror neurons is our demonstration of neuronal tuning to the distance between the passenger and observer. A previous study has already demonstrated that the distance between an observer (a monkey) and an actor (a human performing a motor task in front of the monkey) influences mirror activity in premotor cortex^4^. In our experiments, M1 and PMd neurons in both the passenger and observer were modulated to the distance between the monkeys, especially when the passenger entered the 1-m zone surrounding the observer. Interestingly, swapping the passenger and observer often changed the pattern of this distance tuning, which indicated that the neuronal representation of “someone entering my space” was different from the representation of “me entering someone’s space”.

Our finding of distance-tuning to reward also bears resemblance to previous reports of mirror-like activity related to observation of ingestive actions^61,63–65,71–73^. Here, we demonstrated that both the passenger’s and observer’s M1 and PMd units were modulated to the distance from the passenger to reward. As in the case of tuning to inter-monkey distance, reward-related tuning patterns depended on the monkey pair composition and the assigned monkey roles. This finding suggests that monkey dominance ranks, defined by preferential access to food and aggression^62^, played a role in the cortical encoding of reward location.

Despite being generally consistent with the mirror-neuron framework, our findings clearly go beyond this classical paradigm by describing, for the first time, a potential neuronal mechanism underlying social interaction in the form of episodic ICS involving multiple motor cortical areas. Previously, classic sensorimotor physiology described cortical motor control and associated sensory processing as activities confined to distinct cortical areas located in a single brain. For example, in an individual animal, cortical motor areas would be involved with the planning of an arm movement and then contribute to its execution, while adjusting the movement based on sensory feedback. The finding of mirror neurons adds to this view the notion that the same cortical areas that control movements also respond to the observation of movements performed by others. These descriptions apply to two types of behavior: (1) production of actions, and (2) observation of actions; but do not integrate them. Social interaction is a type of behavior where actions are amalgamated with observations. Accordingly, our discovery that social interaction between pairs of monkeys can be represented by widespread ICS merges both action and observation as part of a common neurophysiological interactive process, taking place simultaneously in the motor cortical areas of multiple primate brains interacting as part of a social group. Therefore, we further propose that studying the patterns of such ICS will increase our knowledge of movements that are planned and executed in the context of the rich social interactions that characterize the lives of primate and other animal groups, including humans. This new view indicates the need to reconsider the role of motor cortical areas to include their involvement in animal social interactions and how the latter influence the moment to moment operation of such cortical motor circuits.

Overall, the present demonstration of neural correlates of social interaction in the form of ICS has implications for future clinical application as well, especially for disorders that include social interaction deficits, such as autism spectrum disorders, which may involve difficulties in representing/understanding the actions of others, while generating appropriate social behaviors^74,75^. Previously, these conditions have been linked to disorders of the brain mirror system^76–79^. Our current findings are relevant to this framework and add to it by showing that social interactions may be encoded by the episodes of ICS. Accordingly, we suggest that our approach could be used as a diagnostic tool for detecting abnormal interbrain activities during human social interactions. In addition to being a potential biomarker for quantifying the severity of different forms of autism, measurement of ICS could be used as a tool for monitoring the effects of autism treatment, like behavior therapy, and also as part of a neurofeedback system for improving social motor skills, like in high-performance collective sports.

## Methods

All animal procedures were performed in accordance with the National Research Council’s Guide for the Care and Use of Laboratory Animals and were approved by the Duke University Institutional Animal Care and Use Committee.

### Study Design

Three adult rhesus macaques (monkey C, K, and J) were used in this study. They were chronically implanted with microwire arrays in multiple cortical areas (monkeys C and K in January 2012, and monkey J in January 2017). In monkey K, we recorded from M1 and PMd; in monkey C, from M1 and PMd; and in monkey J, from M1. Neuronal spiking activity was recorded using a wireless recording system developed in-house that samples from 128 channels simultaneously in each monkey. A pair of monkeys (C and K, or C and J) was placed in the experimental room. One monkey (the observer) sat in a stationary chair while the other monkey (passenger) sat in the robotic wheelchair that navigated in the room. The room size was 5.0-by-3.9 m. The wheelchair navigated in the 3.5-by-2.6 m part of that room. The maximum translational and rotational velocities of the wheelchair were 0.3 m/s and 0.5 rad/s, respectively, to ensure the comfort of the passenger. When placed in the initial position, the passenger faced the observer in one corner of the room and a food dispenser in another corner. The positions of the observer and the food dispenser were swapped in different experiments. After the passenger arrived at the dispenser’s location, the dispenser dropped a piece of fruit (grape, apple, blueberry, or carrot) on a plate mounted on a robotic arm, the arm extended towards the observer, and the observer grasped the food.

### Behavioral task

The robotic wheelchair carried the passenger from a randomly generated starting position toward the food dispenser. The navigation trajectory passed through two randomly generated checkpoints before arriving at the food dispenser location. After the passenger retrieved the food, the wheelchair autonomously navigated back to a new randomly generated starting position. During the wheelchair navigation toward the dispenser, the observer monkey received juice rewards: small drops of juice were delivered every 10–30 s, but only if the observer’s head pointed in the direction the room center. A large juice reward was delivered when the wheelchair arrived at the food dispenser.

### Social ranking

We assessed the social hierarchy in monkey pair C-K and C-J using a paradigm where they competed for food^29–31^. The two monkeys sat in chairs and faced each other at the distance where they could reach for a small piece of fruit (grape, apple, or strawberry) placed on a tray. We conducted 50 trials, where the food was placed in between the monkeys, and we counted how often each of the monkeys reached out first. For the pair C-K, monkey C and K obtained the fruit 32 and 18 times each (${{\rm{\chi }}}^{2}$(1) = 7.8, p < 0.05); for monkey pair C-J, monkey C and J obtained the fruit 36 and 14 times each (${{\rm{\chi }}}^{2}$(1) = 19.4, p < 0.05). These results suggested that monkey C was the dominant monkey in each pair.

### System integration

The experiment setup included three components: (1) the experiment control system, (2) the wheelchair navigation system, and (3) the wireless recording system. The experiment control system supervised the sequence of task events. The autonomous wheelchair navigation system controlled the wheelchair and reported the position of the wheelchair to the experiment control system. The wireless recording system recorded neuronal ensemble activity from two monkey brains simultaneously and sent the spike timestamps to the experiment control system. The three systems communicated using a local network.

#### Experiment Control System

The experiment control system controlled the task sequence, including starting a trial, setting target locations for the wheelchair, determining whether the wheelchair has reached the target, delivering food and liquid rewards, and ending a trial. This system received the wheelchair coordinates from the wheelchair navigation system at 10 Hz and sent target locations to the wheelchair navigation system. The experimental control system also received multichannel neuronal data from the wireless recording system.

#### Wheelchair Navigation System

To move the wheelchair from one location to another, the robust autonomous navigation was implemented. The wheelchair was equipped with a Roboteq VDC2450 dual channel motor controller and wheel encoders to provide closed-loop control and odometry. A lidar (RPLidar 360 Laser Scanner by Robopeak) was installed at the front side of the wheelchair to sense the distance to its surroundings. The motor controller and the lidar were interfaced via a local wired/wireless network with a Raspberry Pi (RP), which communicated with the computer that ran the experiment control system.

We used Robotic Operating System (ROS) software to provide the wheelchair functionality, including autonomous navigation, obstacle avoidance, simultaneous localization and mapping (SLAM). ROS ran on two computers: the RP and a dedicated desktop computer for navigation. The two computers communicated through ROS topics, on which one computer could publish messages and the other could subscribe. To localize the wheelchair, a map of the experiment room (Fig. S3) was first generated by Hector SLAM^80^ before the very first session, and this map was used for all the sessions. Then, combining sensor data published by the RP and wheelchair velocity commands published by the navigation computer, a particle filter approach was used to localize the wheelchair at 10 Hz. Given the position of the wheelchair and the navigation destinations from the experiment control computer, the navigation computer computed and published the wheelchair velocity commands within the predefined ranges (0 to 0.3 m/sec for translations, and −0.5 to 0.5 rad/s for rotations) at 20 Hz (ROS navigation package), which the RP subscribed and passed to the motor controller for execution.

#### Wireless Recording System

The wireless recording system was built in-house, as described in^32,33^. In short, it was composed of two wireless headstages of 128-channels (one for each monkey), two bridge receivers (one for each headstage), and one recording computer. Once spikes were sorted in the recording computer, the 16-point spike waveform templates were transmitted to the storage on the headstage. The headstage sampled neural activities of each channel at 31,250 Hz, and the headstage performed spike sorting on an FPGA using a template algorithm, where a spike was detected if the absolute distance between the waveform and the template was within a user-specified threshold. Detected spike occurrences and channel IDs were wirelessly sent to the bridge receiver and then routed to the recording computer through wired ethernet. The recording computer timed the detected spikes (temporal resolution was estimated about 5 ms) and stored this information and sent neuronal data to the system control computer for further processing, such as online decoding of neural signals.

### Data analysis

We conducted seven sessions with the monkey pair C-K and four sessions with the pair C-J. For C-K, each session lasted 99.3 ± 17.3 (mean ± standard deviation) 99.3 minutes and consisted of 80 ± 45 trials. Only the movements of the wheelchair toward the food dispenser (62.8 ± 10.4 minutes) were analyzed; the returns to the starting positions were excluded from the analysis. Each trial began when the wheelchair started at the starting positions and ended when the wheelchair arrived the target location, where the fruit has not being presented to the monkey at the time so that no reaching related activities was included in the analysis. For the first three experimental sessions, monkey K was the passenger, and monkey C was the passenger during the next three sessions. On the last, the seventh session, monkey K was the passenger again. The observer remained in the same corner of the room for the first five sessions, and then the locations of the observer and grape dispenser were swapped. For the C-J pair, the four sessions lasted on average 55.9 minutes (std. 6.4 minutes) and included 48 trials (std. 1.3 trials). The movements of the wheelchair toward the dispenser lasted 31.5 minutes (std. 0.6 minutes). Monkey C was the passenger for the first two sessions, then monkey J became the passenger for the next two sessions. The observer was located in one room corner for the first and fourth sessions, and the opposite corner for the second and third sessions.

#### Modulation depth

Modulation depth was calculated based on linear regression. We sampled the variates (e.g., wheelchair position) with a 100-ms sampling interval within the interval 500 ms before and after time t and we regressed them to the value of neural firing rate at time t. Once the regression model was trained, we computed correlation coefficient between the true and the fitted firing rate time series as the uncorrected modulation depth (*r*′).

To test whether a unit was significantly modulated to a variate, we compared $r^{\prime}$ against the modulation depth $r^{\prime\prime}$ computed from randomly sampled and permuted firing rate series. We performed the permutation test with 1,000 permutations, and the p-values were corrected by false-discovery rate. We considered a unit significantly modulated if the corrected p-value was under 0.05. Note that for each ICS condition, only the firing rate time series that belonged to that condition was permuted and tested against.

To compare neuronal modulation under different ICS conditions, the modulation depth r was computed from the same length of data across conditions, where the length was the duration of synchronized episodes for the whole session. Finally, we computed unbiased modulation depth, $r=r^{\prime} -mean(r^{\prime\prime} )$, $r$ was used as the metric for modulation depth in all analyses.

#### ANOVA for modulation depth

We assessed the effect of the presence of synchrony episodes (ICS+ vs. ICS−), monkey role (passenger vs. observer), social rank (dominant vs. subordinate), and monkey pair (C-K vs. C-J) on modulation depth using a mixed-designed four-way ANOVA, where ICS episode was a repeated measure obtained from the same unit, while others were non-repeated measures. Modulation depth of each unit was treated as the random effect, while all others were fixed effect. Sums of squares of type 3 were employed when the data was unbalanced.

#### ANOVA for firing rates

ANOVA was designed the same way as that for modulation depth.

#### ICS metric

Bias-corrected distance correlation was originally proposed by Székely and Rizzo^81^ to quantify the strength of correlation between two random vectors in arbitrarily high dimensions that do not need to be equal. We chose distance correlation over RV coefficient because distance correlation is more sensitive to detect nonlinear dependency^82^. The distance correlation was computed as ${{\rm{R}}}_{{\rm{n}}}^{\ast }({\rm{X}},\,{\rm{Y}})=\frac{{{\rm{V}}}_{{\rm{n}}}^{\ast }({\rm{X}},{\rm{Y}})}{\sqrt{{{\rm{V}}}_{{\rm{n}}}^{\ast }({\rm{X}},{\rm{X}}){{\rm{V}}}_{{\rm{n}}}^{\ast }({\rm{Y}},{\rm{Y}})}}$, where ${{\rm{V}}}_{{\rm{n}}}^{\ast }({\rm{X}},{\rm{Y}})$ is the modified distance covariance between two random vectors ${\rm{X}}\in {{\mathbb{R}}}^{{\rm{p}}}$ and ${\rm{Y}}\in {{\mathbb{R}}}^{{\rm{q}}}$. This distance correlation test statistic has an asymptotic student t distribution under independence, and thus t-test is used for multivariate independence in high dimension. Also, ${{\rm{R}}}_{{\rm{n}}}^{\ast }$ is always positive if ${\rm{X}}$ and ${\rm{Y}}$ are correlated.

Brain-to-brain correlation was computed as bias-corrected distance correlation between two random vectors, where each random vector was the neural firing rate (0.1 s time bins) of the population of units from each monkey. When analyzing episodic brain-to-brain correlation, the correlation was computed within a 3-s sliding window shifted with 0.1 s steps.

#### Episodic ICS

To test whether an ICS episode was significant, we first computed interbrain correlation for all 0.1 s time steps (see ICS metric above). Then, we permuted the spike trains from one monkey and computed the correlation again for the permuted data, which resulted in a null distribution of ICS (Fig. S1). Lastly, we tested ICS value of each 3 s sliding window against this null distribution to determine whether it is a significant ICS episode (t-test with alpha = 0.05 for a right-tail test, and p-value was corrected by false discovery rate).

#### ICS Probability

ICS probability was calculated as the conditional probability of the occurrence of an ICS episode given the wheelchair position or velocity. To compute the ICS probability as a function of room coordinates of the wheelchair, the room coordinates were binned into a 0.2 m^2^ grid, where x spanned from 0.1 m to 3.1 m and y spanned from −0.8 m to 1.2 m. The ICS probability was computed for each bin as the count of ICS episodes, divided by the total count of the wheelchair entering the bin. Similarly, the passenger-grape distance and the passenger-observer distance were both binned from 0.4 m to 4 m with 0.2 m step. To compute the ICS probability as a function of the wheelchair kinematics, the rotational velocity was binned from −0.5 to 0.5 rad/s with bin size of 0.05 rad/s; the translational velocity was binned from 0 to 0.3 m/s with bin size of 0.05 m/s.

#### Decoder output correlation

Correlation between the decoder outputs was computed as the bias-corrected distance correlation between the decoded velocities decoded for each monkey. This correlation was also computed within 3-s sliding window shift with 0.1 s steps.

#### Flip-index

Flip-index measured if the neuronal modulation pattern to different room locations symmetrically reflected when the locations of the food dispenser and the observer were swapped. We first computed pixel-wise Spearman correlation between the spatial modulation patterns being compared. Then, we flipped one of the patterns and computed the correlation coefficient again. The flip index of a unit was the difference between these two correlation coefficient values. Note that, because the food dispenser and the observer only swapped locations between different sessions, we assumed units of the same ID number from different sessions were the same units for this analysis (see Supplementary Fig. S5). Also, the flip index was calculated between sessions where the monkeys had the same roles.

#### ANOVA for flip-index

Multi-factor ANOVA assessed the effect of the presence of synchrony episodes (ICS+ vs. ICS−), monkey role (passenger vs. observer), recorded area (M1 vs. PMd), and social rank (dominant vs. subordinate) on flip-index. We used a mixed-designed four-way ANOVA. We treated synchrony episodes as repeated measures from the same units, while the others as non-repeated measures. Flip-index was the only random effect, while others were fixed effect. Sums of squares of type 3 were employed when the data was unbalanced.

## Electronic supplementary material

Supplementary Materials

## References

[1] Cook R, Bird G, Catmur C, Press C, Heyes C (2014). Mirror neurons: from origin to function. Behav. Brain Sci..

[2] Dunbar RIM, Shultz S (2007). Evolution in the Social Brain. Science (80-)..

[3] Caggiano V (2012). Mirror neurons encode the subjective value of an observed action. Proc. Natl. Acad. Sci..

[4] Caggiano V, Fogassi L, Rizzolatti G, Thier P, Casile A (2009). Mirror neurons differentially encode the peripersonal and extrapersonal space of monkeys. Science (80-)..

[5] di Pellegrino G, Fadiga L, Fogassi L, Gallese V, Rizzolatti G (1992). Understanding motor events: a neurophysiological study. Exp. brain Res..

[6] Dushanova J, Donoghue J (2010). Neurons in primary motor cortex engaged during action observation. Eur. J. Neurosci..

[7] Fujii, N., Hihara, S. & Iriki, A. Dynamic social adaptation of motion-related neurons in primate parietal cortex. *PLoS One* **2** (2007). 10.1371/journal.pone.0000397 PMC1851098 17460764

[8] Gallese V, Fadiga L, Fogassi L, Rizzolatti G (1996). Action recognition in the premotor cortex. Brain.

[9] Ifft PJ, Shokur S, Li Z, Lebedev MA, Nicolelis MAL (2013). A brain-machine interface enables bimanual arm movements in monkeys. Sci. Transl. Med.

[10] Ishida H, Nakajima K, Inase M, Murata A (2010). Shared mapping of own and others’ bodies in visuotactile bimodal area of monkey parietal cortex. J. Cogn. Neurosci..

[11] Kraskov A, Dancause N, Quallo MM, Shepherd S, Lemon RN (2009). Corticospinal neurons in macaque ventral premotor cortex with mirror properties: a potential mechanism for action suppression?. Neuron.

[12] O’Doherty JE (2011). Active tactile exploration using a brain-machine-brain interface. Nature.

[13] Shepherd SV, Klein JT, Deaner RO, Platt ML (2009). Mirroring of attention by neurons in macaque parietal cortex. Proc. Natl. Acad. Sci..

[14] Yoshida K, Saito N, Iriki A, Isoda M (2011). Representation of others’ action by neurons in monkey medial frontal cortex. Curr. Biol..

[15] Rizzolatti G, Cattaneo L, Fabbri-Destro M, Rozzi S (2014). Cortical mechanisms underlying the organization of goal-directed actions and mirror neuron-based action understanding. Physiol. Rev..

[16] Fabbri-Destro M, Rizzolatti G (2008). Mirror Neurons and Mirror Systems in Monkeys and Humans. Physiology.

[17] Ferrari PF, Rozzi S, Fogassi L (2005). Mirror Neurons Responding to Observation of Actions Made with Tools in Monkey Ventral PremotorCortex. J. Cogn. Neurosci.

[18] Gallese V, Keysers C, Rizzolatti G (2004). A unifying view of the basis of social cognition. Trends in Cognitive Sciences.

[19] Heyes C (2010). Where do mirror neurons come from?. Neurosci. Biobehav. Rev..

[20] Iacoboni M (1999). Cortical mechanisms of human imitation. Science.

[21] Oberman LM, Pineda JA, Ramachandran VS (2007). The human mirror neuron system: A link between action observation and social skills. Soc. Cogn. Affect. Neurosci..

[22] Ramachandran, V. S. Mirror neurons and imitation learning as the driving force behind ‘the great leap forward’ in human evolution. (2000).

[23] Haroush K, Williams ZM (2015). Neuronal prediction of opponent’s behavior during cooperative social interchange in primates. Cell.

[24] Yoshida K, Saito N, Iriki A, Isoda M (2012). Social error monitoring in macaque frontal cortex. Nat. Neurosci..

[25] Altmann S (1965). a. Sociobiology of rhesus monkeys. II. Stochastics of social communication. J. Theor. Biol..

[26] Lauer C (1980). Seasonal variability in spatial defence by free-ranging rhesus monkeys (Macaca mulatta). Anim. Behav..

[27] Mitani JC, Rodman PS (1979). Territoriality: The relation of ranging pattern and home range size to defendability, with an analysis of territoriality among primate species. Behav. Ecol. Sociobiol..

[28] van Schaik CP, van Noordwijk MA, de Boer RJ, den Tonkelaar I (1983). The effect of group size on time budgets and social behaviour in wild long-tailed macaques (Macaca fascicularis). Behav. Ecol. Sociobiol..

[29] Belzung C, Anderson JR (1986). Social rank and responses to feeding competition in rhesus monkeys. Behav. Processes.

[30] Brennan J, Anderson JR (1988). Varying responses to feeding competition in a group of rhesus monkeys (Macaca mulatta). Primates.

[31] Deutsch JC, Lee PC (1991). Dominance and feeding competition in captive rhesus monkeys. Int. J. Primatol..

[32] Rajangam S (2016). Wireless Cortical Brain-Machine Interface for Whole-Body Navigation in Primates. Sci. Rep..

[33] Schwarz DA (2014). Chronic, wireless recordings of large-scale brain activity in freely moving rhesus monkeys. Nat. Methods.

[34] Allison T, Puce A, McCarthy G (2000). Social perception from visual cues: Role of the STS region. Trends in Cognitive Sciences.

[35] De Gelder B (2006). Towards the neurobiology of emotional body language. Nature Reviews Neuroscience.

[36] Jellema T, Perrett DI (2003). Cells in monkey STS responsive to articulated body motions and consequent static posture: a case of implied motion?. Neuropsychologia.

[37] Mazur A (1985). A biosocial model of status in face-to-face primate groups. Soc. Forces.

[38] Dikker S (2017). Brain-to-Brain Synchrony Tracks Real-World Dynamic Group Interactions in the Classroom. Curr. Biol..

[39] Gollo, L. L., Mirasso, C., Sporns, O. & Breakspear, M. Mechanisms of Zero-Lag Synchronization in Cortical Motifs. *PLoS Comput. Biol*. **10**, (2014). 10.1371/journal.pcbi.1003548 PMC3998884 24763382

[40] Hasson U, Ghazanfar AA, Galantucci B, Garrod S, Keysers C (2012). Brain-to-brain coupling: A mechanism for creating and sharing a social world. Trends Cogn. Sci..

[41] Nijholt, A. In *Brain-Computer Interfaces* **74**, 313–335 (Springer, 2015).

[42] Schilbach L (2013). Toward a second-person neuroscience. Behav. Brain Sci..

[43] Montague PR (2002). Hyperscanning: simultaneous fMRI during linked social interactions. Neuroimage.

[44] Anders S, Heinzle J, Weiskopf N, Ethofer T, Haynes JD (2011). Flow of affective information between communicating brains. Neuroimage.

[45] King-Casas B (2005). Getting to know you: reputation and trust in a two-person economic exchange. Science.

[46] Schippers MB, Gazzola V, Goebel R, Keysers C (2009). Playing charades in the fMRI: Are mirror and/or mentalizing areas involved in gestural communication?. PLoS One.

[47] Schippers MB, Roebroeck A, Renken R, Nanetti L, Keysers C (2010). Mapping the information flow from one brain to another during gestural communication. Proc. Natl. Acad. Sci. USA.

[48] Tomlin D (2006). Agent-specific responses in the cingulate cortex during economic exchanges. Science.

[49] Stephens GJ, Silbert LJ, Hasson U (2010). Speaker-listener neural coupling underlies successful communication. Proc. Natl. Acad. Sci..

[50] Lee, R. F. Emergence of the default-mode network from resting-state to activation-state in reciprocal social interaction via eye contact. *Proc. Annu. Int. Conf. IEEE Eng. Med. Biol. Soc. EMBS* 2015–Novem, 1821–1824 (2015). 10.1109/EMBC.2015.7318734 26736634

[51] Fliessbach K (2007). Social comparison affects reward-related brain activity in the human ventral striatum. Science.

[52] Bilek E (2015). Information flow between interacting human brains: Identification, validation, and relationship to social expertise. Proc. Natl. Acad. Sci. USA.

[53] Lee RF, Dai W, Dix W (2010). A decoupled circular-polarized volume head coil pair for studying two interacting human brains with MRI. Conf. Proc. IEEE Eng. Med. Biol. Soc.

[54] Lee RF, Dai W, Jones J (2012). Decoupled circular-polarized dual-head volume coil pair for studying two interacting human brains with dyadic fMRI. Magn. Reson. Med..

[55] Ramakrishnan A (2015). Computing Arm Movements with a Monkey Brainet. Sci. Rep..

[56] Rosenboom, D. *Biofeedback and the arts: results of early experiments*. (Aesthetic Research Centre of Canada: Vancouver, B.C., 1976).

[57] Rosenboom D (1990). The Performing Brain. Comput. Music J..

[58] Sobell N, Trivich M (1989). Brainwave drawing game. Delicate Balance: Technics, Culture and Consequences.

[59] Brody EB, Rosvold EH (1952). Influence of prefrontal lobotomy on social interaction in a monkey group. Psychosom. Med..

[60] Sahlins MD (1959). The social life of monkeys, apes and primitive man. Hum. Biol..

[61] Ferrari PF, Maiolini C, Addessi E, Fogassi L, Visalberghi E (2005). The observation and hearing of eating actions activates motor programs related to eating in macaque monkeys. Behav. Brain Res..

[62] Harlow HF, Yudin HC (1933). Social behavior of primates. I. Social facilitation of feeding in the monkey and its relation to attitudes of ascendance and submission. J. Comp. Psychol..

[63] Marsh BT, Tarigoppula VSA, Chen C, Francis JT (2015). Toward an autonomous brain machine interface: integrating sensorimotor reward modulation and reinforcement learning. J. Neurosci..

[64] Ramakrishnan A (2017). Cortical neurons multiplex reward-related signals along with sensory and motor information. Proc. Natl. Acad. Sci..

[65] Ramkumar P, Dekleva B, Cooler S, Miller L, Kording K (2016). Premotor and Motor Cortices Encode Reward. PLoS One.

[66] Darby CL, Riopelle AJ (1959). Observational learning in the rhesus monkey. J. Comp. Physiol. Psychol..

[67] Petrosini L (2003). Watch how to do it! New advances in learning by observation. Brain Res. Brain Res. Rev..

[68] Fujii N, Hihara S, Nagasaka Y, Iriki A (2009). Social state representation in prefrontal cortex. Soc. Neurosci..

[69] Oosugi N, Yanagawa T, Nagasaka Y, Fujii N (2016). Social Suppressive Behavior Is Organized by the Spatiotemporal Integration of Multiple Cortical Regions in the Japanese Macaque. PLoS One.

[70] Lebedev MA, Wise SP (2001). Tuning for the orientation of spatial attention in dorsal premotor cortex. Eur. J. Neurosci..

[71] Ferrari PF, Gallese V, Rizzolatti G, Fogassi L (2003). Mirror neurons responding to the observation of ingestive and communicative mouth actions in the monkey ventral premotor cortex. Eur. J. Neurosci..

[72] Haruno M, Kawato M (2006). Different neural correlates of reward expectation and reward expectation error in the putamen and caudate nucleus during stimulus-action-reward association learning. J. Neurophysiol..

[73] Hollerman JR, Tremblay L, Schultz W (1998). Influence of reward expectation on behavior-related neuronal activity in primate striatum. J. Neurophysiol..

[74] Frith U, Happé F, Happe F (2005). Autism spectrum disorder. Curr. Biol..

[75] Lord C, Cook EH, Leventhal BL, Amaral DG (2000). Autism spectrum disorders. Neuron.

[76] Dapretto M (2006). Understanding emotions in others: mirror neuron dysfunction in children with autism spectrum disorders. Nat. Neurosci..

[77] Oberman LM (2005). EEG evidence for mirror neuron dysfunction in autism spectrum disorders. Cogn. Brain Res..

[78] Perkins T, Stokes M, McGillivray J, Bittar R (2010). Mirror neuron dysfunction in autism spectrum disorders. J. Clin. Neurosci..

[79] Théoret H (2005). Impaired motor facilitation during action observation in individuals with autism spectrum disorder. Curr. Biol..

[80] Kohlbrecher, S., Von Stryk, O., Meyer, J. & Klingauf, U. A flexible and scalable SLAM system with full 3D motion estimation. *9th IEEE Int. Symp. Safety, Secur. Rescue Robot. SSRR* **2011** 155–160, 10.1109/SSRR.2011.6106777 (2011).

[81] Székely GJ, Rizzo ML (2013). The distance correlation t -test of independence in high dimension. J. Multivar. Anal..

[82] Josse J, Holmes S (2013). Measures of dependence between random vectors and tests of independence. Literature review..

