# Supplementary material for: Interbrain cortical synchronization encodes multiple aspects of social interactions in monkey pairs
Source: Sci Rep. 2018 Mar 29;8:4699. doi: 10.1038/s41598-018-22679-x (PMC5876380; doi:10.1038/s41598-018-22679-x)
Supplement: Supplementary file 1 — Supplementary Materials [file 41598_2018_22679_MOESM1_ESM.docx]

Supplementary Materials

Interbrain cortical synchronization encodes multiple aspects of social interactions in monkey pairs

Po-He Tseng^1,2^, Sankaranarayani Rajangam^1,2^, Gary Lehew^1,2^, Mikhail A. Lebedev^1,2^,

Miguel A.L. Nicolelis^1^*^,2,3,4,5,6^

^1^Department of Neurobiology, Duke University.

^2^Duke University Center for Neuroengineering, Duke University.

^3^Department of Biomedical Engineering, Duke University

^4^Department of Psychology and Neuroscience, Duke University

^5^Department of Neurology, Duke University

^6^Edmund and Lily Safra International Institute of Neurosciences, Natal 59066060, Brazil

***Corresponding Author:**

Miguel A.L. Nicolelis, MD, PhD

Box 103905 Duke University

Durham, NC 27710

Phone: 919-668-6031 Email: [nicoleli@neuro.duke.edu](mailto:nicoleli@neuro.duke.edu)

**Controlling firing rate in ICS**

The population-median firing rate was 28.04 spikes/s during ICS and 24.78 spikes/s in the absence of ICS, and the median for the change from ICS to no-ICS for each unit was 1.20 spikes/s. To test whether this difference in firing rate affected the percentage of units modulated to the wheelchair velocity (and position), we conducted an analysis where we randomly removed or added spikes from the ICS periods to equalize absolute rates during and outside ICS for each unit (permutation test of 1000 permutations, p>0.83). Next, we performed the analysis that determined the percentage of modulated units. We found no change compared to the original analysis for all monkeys, and for both velocity and position (χ2(1)<1.42, p>0.23).

**Supplementary Figures**


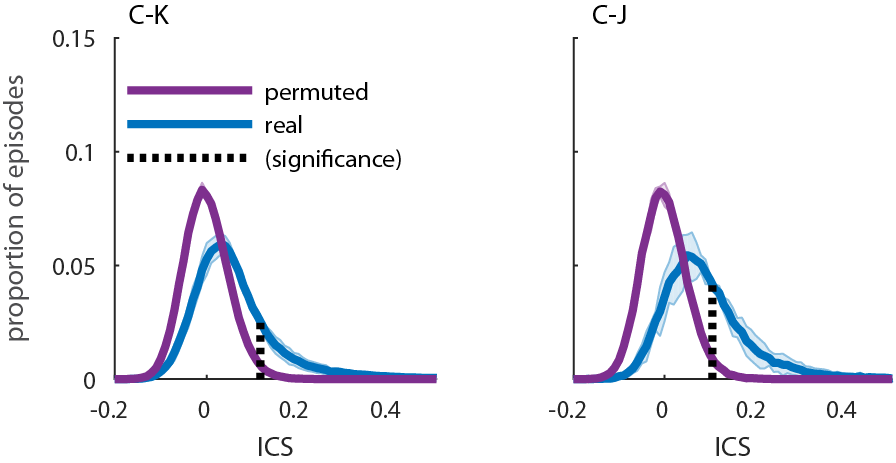


**Figure S1. Statistical distribution of the correlation coefficient for ICS.** The panel on the left corresponds to monkey pair C-K, and the panel on the right to monkey pair C-J. Correlation coefficient values were calculated for the data within a 3-s sliding window. They represented bias-corrected distance correlation of firing rates between the cortical ensembles of two monkeys. The permuted distributions were obtained by permuting the data points for one monkey. Shaded regions represent 95% confidence interval obtained by 1,000 bootstrap replicates. The vertical dash lines represent the statistical criterion that defines inside and outside of interbrain cortical synchronization. Defined by this criterion, ICS episodes constituted 19.7%±2.1% (mean±standard error) of the total session time with a correlation coefficient of 0.208±0.002 for monkey pair C-K, and 35.7%±4.7% with a correlation coefficient of 0.18.5±0.005 for C-J.

*
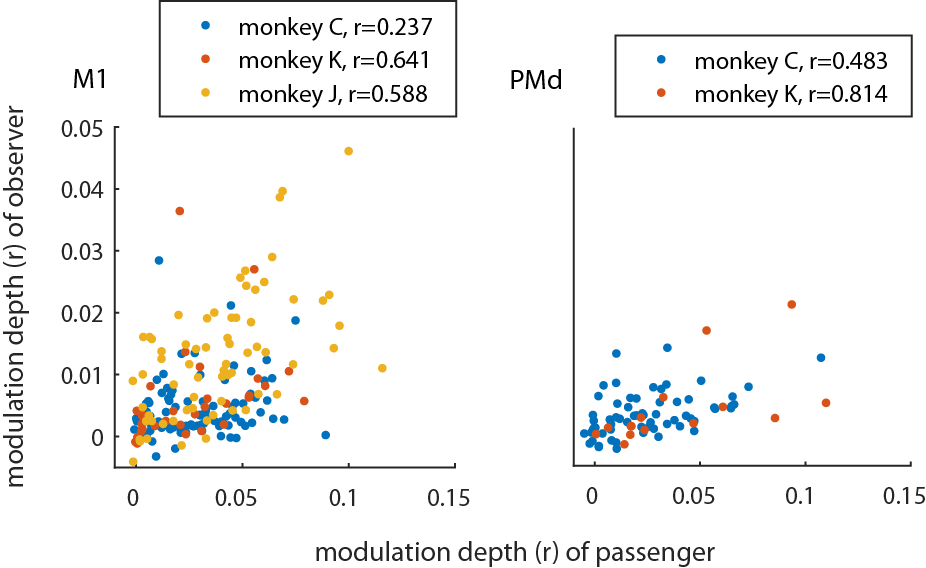
*

**Figure S2. Changes in neuronal modulation depth after passenger became observer.** Each dot corresponds to a unit. Dot color corresponds to the monkey (C, K, or J; see insets). The horizontal axis represents modulation depth for the passenger, and the vertical axis represents observer’s modulation depth. Correlation between the passenger’s and the observer’s modulation depths was evaluated as a Spearman correlation coefficient, r, listed separately for each cortical area and each monkey. Results for M1 are shown on the left, and for PMd on the right.

*
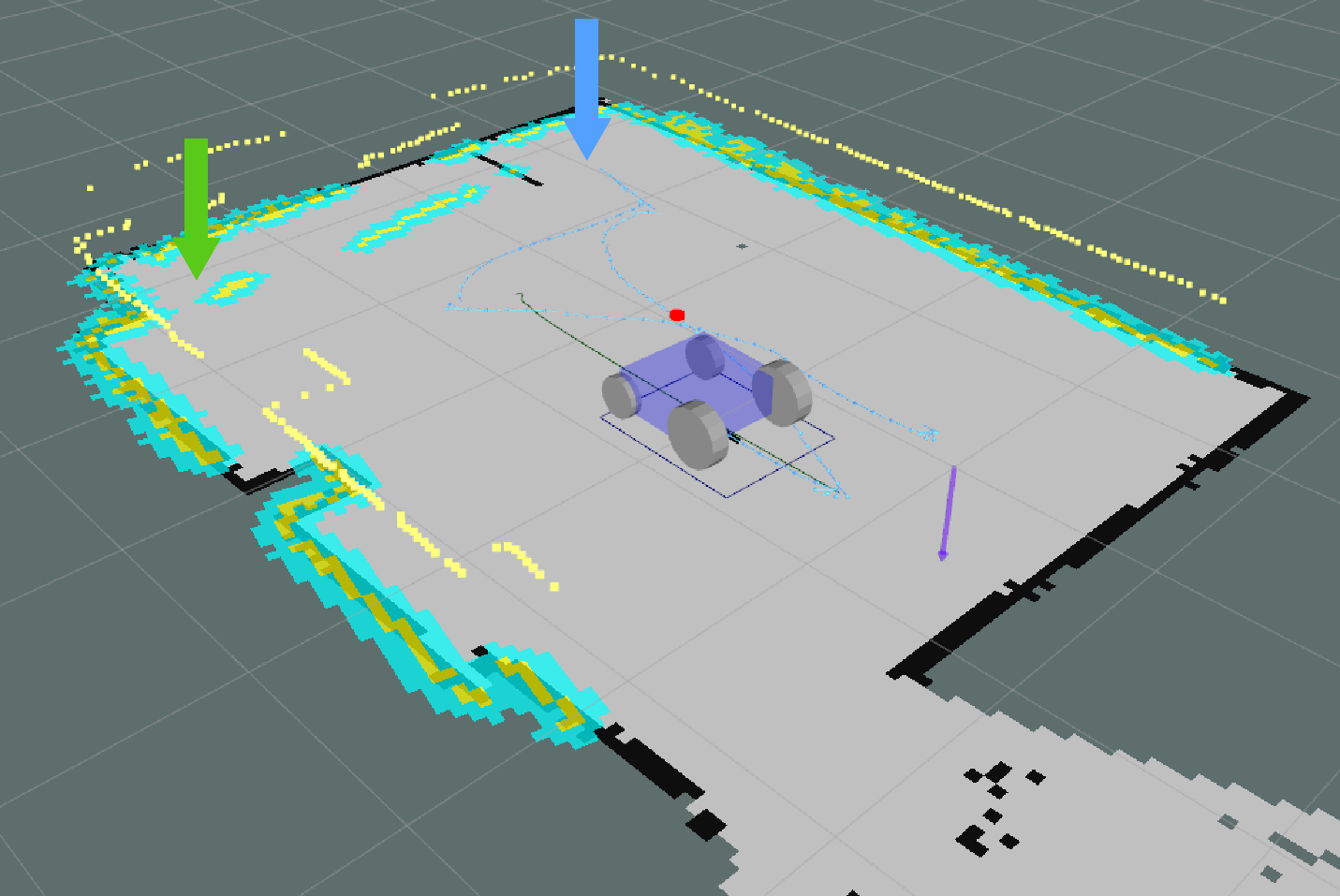
*

**Fig S3. A screenshot from the ROS rviz package showing the experiment room and the wheelchair.** Smaller wheels correspond to the front side of the wheelchair. The yellow dots are the readings from the wheelchair lidar (red disk) that detected walls and obstacles. The grid size is 1-by-1 m. The green arrow indicates the location of the observer, and the blue arrow represents the location that the passenger retrieves grape reward.


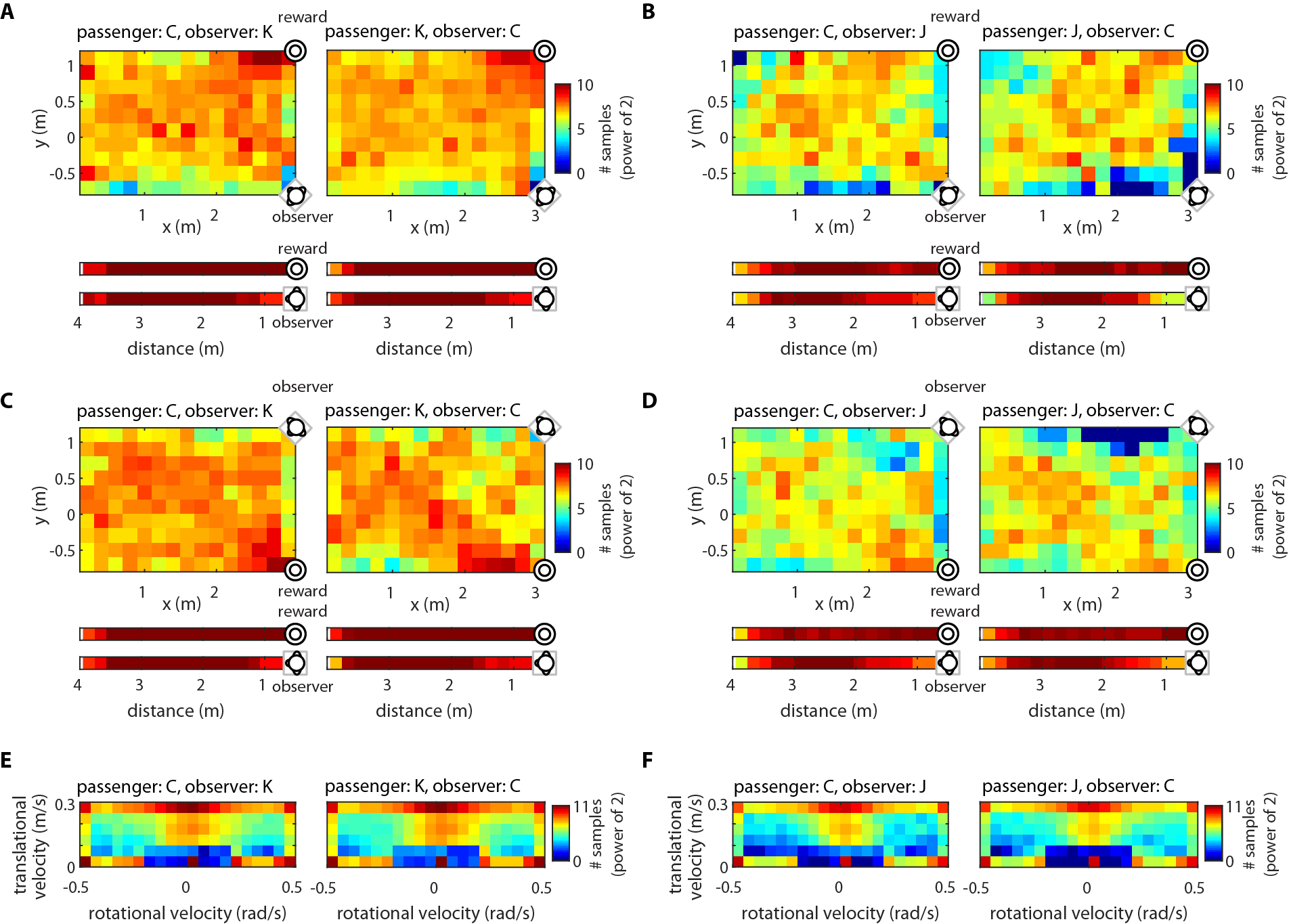


**Fig S4. Number of samples (log_2_ scale) that contributed to heatmaps of Fig. 2.** Panels and conventions are the same as in Fig. 2.


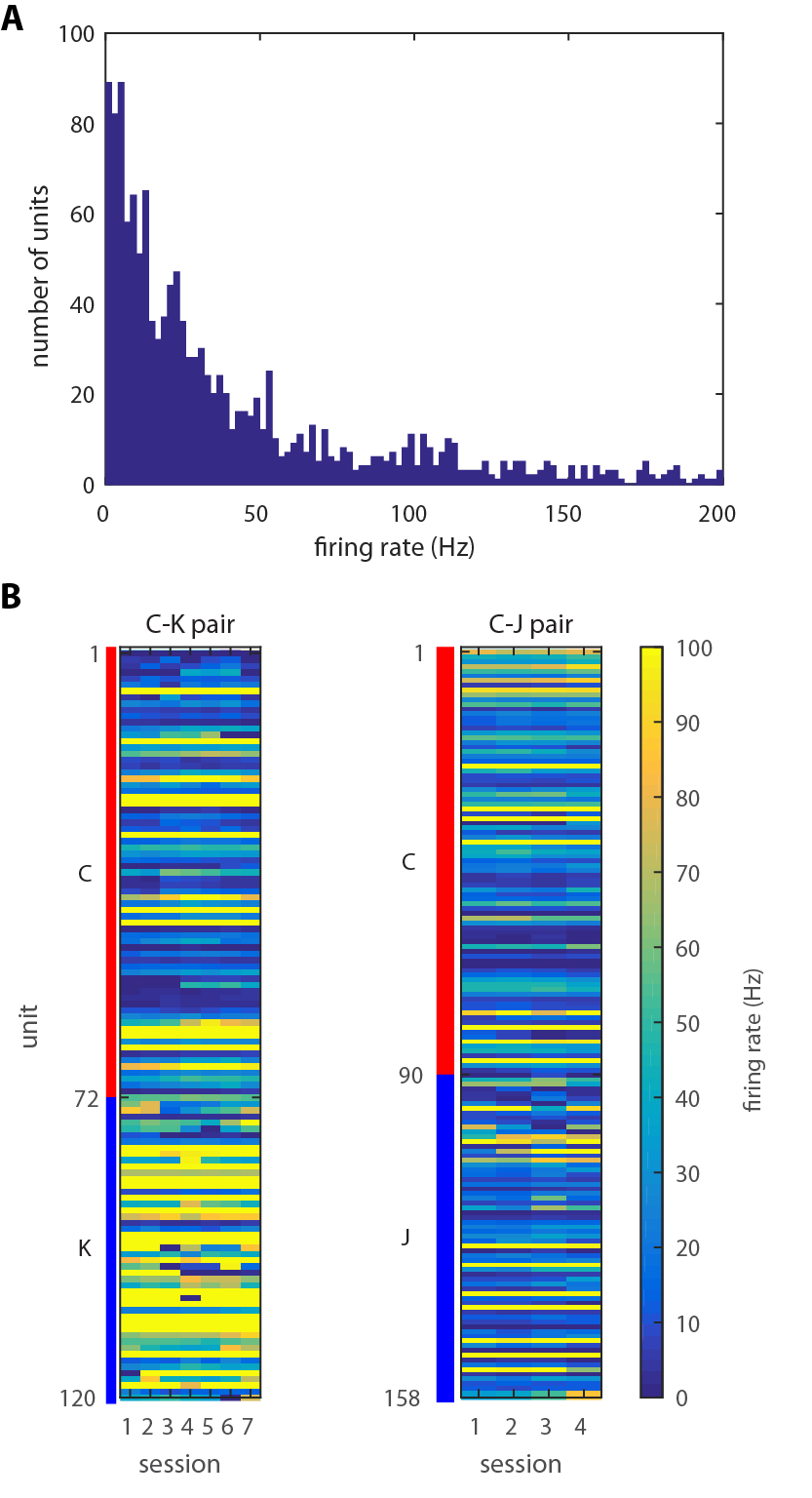


**Fig S5. Absolute firing rates for different units.** (A) Statistical distribution of firing rates for all the units recorded. Fifty percent of the units had average firing rate lower than 25.5Hz, while the average rate for the upper quartile of the distribution was 68.0 Hz. The tail of the distribution corresponds to multiunit activity with high firing rate (up to 200Hz). (B) Daily changes in firing rates. Each row is a unit, and each column is a session. Color corresponds to average firing rate. The plot on the left corresponds to monkey pair C-K, and the plot on the right corresponds to pair C-J. Overall, the distribution of firing rates across different units changed little over several days.


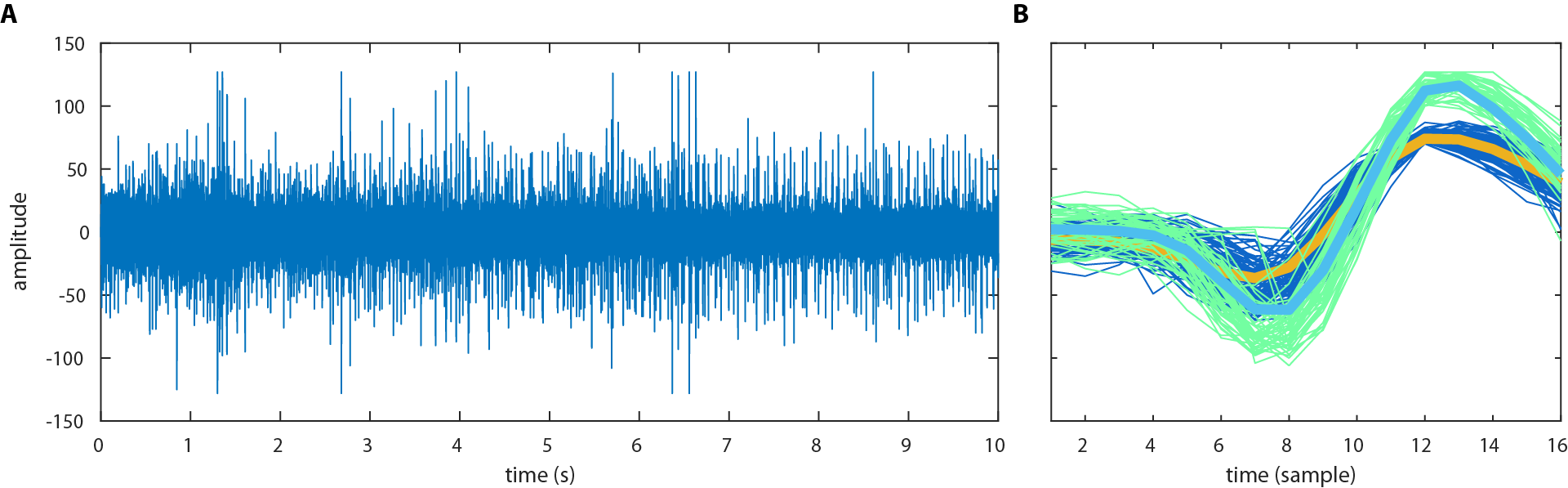


**Fig S6. A representative recording channel.** (A) A 10s snippet for a representative recording channel. (B) Spike waveforms of two sorted units from the same channel. Due to the wireless recording bandwidth, raw waveforms were recorded from only four channels per recording session. For these channels, we conducted offline sorting and computed signal-to-noise ratio (SNR): the peak-to-valley amplitude of the 16-point spike waveform divided by the peak-to-valley amplitude of 16 points just before the waveform. The average SNR was 4.46±0.30 from all the channels available.

**Supplementary Tables**

**Table S1.** ANOVA table for testing modulation depth to the wheelchair velocity. Effect, ANOVA factors and interactions; DFn, degrees of freedom in the numerator; DFd, degrees of freedom in the denominator; F, F-value; p, p-value; p<.05, highlights p-values less than the alpha level of .05; ges, generalized eta-squared measure of effect size; rank, dominant or subordinate monkey; role, passenger or observer; pair, C-K or C-J pair; ics, presence or absence of synchrony.

| Effect | DFn | DFd | F | p | p<.05 | ges |
| --- | --- | --- | --- | --- | --- | --- |
| rank | 1 | 1434 | 13.56539 | 0.00024 | * | 0.00621 |
| role | 1 | 1434 | 287.0747 | 0.00000 | * | 0.11672 |
| pair | 1 | 1434 | 9.30229 | 0.00233 | * | 0.00426 |
| ics | 1 | 1434 | 7.572745 | 0.00600 | * | 0.00179 |
| rank:role | 1 | 1434 | 5.977531 | 0.01461 | * | 0.00274 |
| rank:pair | 1 | 1434 | 4.574122 | 0.03263 | * | 0.00210 |
| role:pair | 1 | 1434 | 3.208501 | 0.07347 |  | 0.00147 |
| rank:ics | 1 | 1434 | 12.32696 | 0.00046 | * | 0.00291 |
| role:ics | 1 | 1434 | 2.895566 | 0.08904 |  | 0.00069 |
| pair:ics | 1 | 1434 | 0.179198 | 0.67213 |  | 0.00004 |
| rank:role:pair | 1 | 1434 | 0.156782 | 0.69220 |  | 0.00007 |
| rank:role:ics | 1 | 1434 | 0.012195 | 0.91208 |  | 0.00000 |
| rank:pair:ics | 1 | 1434 | 2.59669 | 0.10731 |  | 0.00062 |
| role:pair:ics | 1 | 1434 | 5.580504 | 0.01829 | * | 0.00132 |
| rank:role:pair:ics | 1 | 1434 | 25.021 | 0.00000 | * | 0.00590 |

**Table S2.** ANOVA table for testing modulation depth to the wheelchair acceleration. See Table S1 for abbreviations.

| Effect | DFn | DFd | F | p | p<.05 | ges |
| --- | --- | --- | --- | --- | --- | --- |
| rank | 1 | 1434 | 0.06006 | 0.80644 |  | 0.00003 |
| role | 1 | 1434 | 270.22010 | 0.00000 | * | 0.10125 |
| pair | 1 | 1434 | 0.39024 | 0.53227 |  | 0.00016 |
| ics | 1 | 1434 | 0.03021 | 0.86203 |  | 0.00001 |
| rank:role | 1 | 1434 | 0.34094 | 0.55938 |  | 0.00014 |
| rank:pair | 1 | 1434 | 0.51526 | 0.47299 |  | 0.00021 |
| role:pair | 1 | 1434 | 0.01682 | 0.89682 |  | 0.00001 |
| rank:ics | 1 | 1434 | 1.67219 | 0.19617 |  | 0.00047 |
| role:ics | 1 | 1434 | 2.56591 | 0.10941 |  | 0.00072 |
| pair:ics | 1 | 1434 | 3.89949 | 0.04849 | * | 0.00109 |
| rank:role:pair | 1 | 1434 | 0.11717 | 0.73217 |  | 0.00005 |
| rank:role:ics | 1 | 1434 | 3.41174 | 0.06494 |  | 0.00096 |
| rank:pair:ics | 1 | 1434 | 0.00141 | 0.97010 |  | 0.00000 |
| role:pair:ics | 1 | 1434 | 0.00709 | 0.93289 |  | 0.00000 |
| rank:role:pair:ics | 1 | 1434 | 16.16731 | 0.00006 | * | 0.00451 |

**Table S3.** ANOVA table for testing modulation depth to the wheelchair position. See Table S1 for abbreviations.

| Effect | DFn | DFd | F | p | p<.05 | ges |
| --- | --- | --- | --- | --- | --- | --- |
| rank | 1 | 1434 | 8.63048 | 0.00336 | * | 0.00473 |
| role | 1 | 1434 | 88.06885 | 0.00000 | * | 0.04624 |
| pair | 1 | 1434 | 1.46903 | 0.22570 |  | 0.00081 |
| ics | 1 | 1434 | 31.80901 | 0.00000 | * | 0.00465 |
| rank:role | 1 | 1434 | 16.32957 | 0.00006 | * | 0.00891 |
| rank:pair | 1 | 1434 | 5.24007 | 0.02222 | * | 0.00288 |
| role:pair | 1 | 1434 | 3.24559 | 0.07183 |  | 0.00178 |
| rank:ics | 1 | 1434 | 0.02297 | 0.87956 |  | 0.00000 |
| role:ics | 1 | 1434 | 13.73573 | 0.00022 | * | 0.00201 |
| pair:ics | 1 | 1434 | 0.29042 | 0.59004 |  | 0.00004 |
| rank:role:pair | 1 | 1434 | 4.80523 | 0.02853 | * | 0.00264 |
| rank:role:ics | 1 | 1434 | 6.78560 | 0.00928 | * | 0.00100 |
| rank:pair:ics | 1 | 1434 | 6.79327 | 0.00925 | * | 0.00100 |
| role:pair:ics | 1 | 1434 | 2.35660 | 0.12497 |  | 0.00035 |
| rank:role:pair:ics | 1 | 1434 | 6.41136 | 0.01145 | * | 0.00094 |

**Table S4.** ANOVA table for testing modulation depth to the observer-passenger distance. See Table S1 for abbreviations.

| Effect | DFn | DFd | F | p | p<.05 | ges |
| --- | --- | --- | --- | --- | --- | --- |
| rank | 1 | 1434 | 12.14626 | 0.00051 | * | 0.00597 |
| role | 1 | 1434 | 7.20403 | 0.00736 | * | 0.00355 |
| pair | 1 | 1434 | 2.35180 | 0.12536 |  | 0.00116 |
| ics | 1 | 1434 | 11.64511 | 0.00066 | * | 0.00236 |
| rank:role | 1 | 1434 | 6.80521 | 0.00918 | * | 0.00335 |
| rank:pair | 1 | 1434 | 17.45884 | 0.00003 | * | 0.00855 |
| role:pair | 1 | 1434 | 17.55769 | 0.00003 | * | 0.00860 |
| rank:ics | 1 | 1434 | 4.37668 | 0.03661 | * | 0.00089 |
| role:ics | 1 | 1434 | 1.94242 | 0.16362 |  | 0.00039 |
| pair:ics | 1 | 1434 | 0.83096 | 0.36215 |  | 0.00017 |
| rank:role:pair | 1 | 1434 | 62.79595 | 0.00000 | * | 0.03010 |
| rank:role:ics | 1 | 1434 | 5.88873 | 0.01536 | * | 0.00120 |
| rank:pair:ics | 1 | 1434 | 0.91818 | 0.33811 |  | 0.00019 |
| role:pair:ics | 1 | 1434 | 0.13534 | 0.71301 |  | 0.00003 |
| rank:role:pair:ics | 1 | 1434 | 5.14861 | 0.02341 | * | 0.00105 |

**Table S5.** ANOVA table for testing neuronal firing rates when the observer-passenger distance was less than 1m. See Table S1 for abbreviations.

| Effect | DFn | DFd | F | p | p<.05 | ges |
| --- | --- | --- | --- | --- | --- | --- |
| rank | 1 | 1434 | 7.22229 | 0.00728 | * | 0.00360 |
| role | 1 | 1434 | 43.60147 | 0.00000 | * | 0.02132 |
| pair | 1 | 1434 | 18.49851 | 0.00002 | * | 0.00916 |
| ics | 1 | 1434 | 358.68020 | 0.00000 | * | 0.06622 |
| rank:role | 1 | 1434 | 7.04481 | 0.00804 | * | 0.00351 |
| rank:pair | 1 | 1434 | 0.53065 | 0.46645 |  | 0.00027 |
| role:pair | 1 | 1434 | 43.73453 | 0.00000 | * | 0.02138 |
| rank:ics | 1 | 1434 | 1.10449 | 0.29346 |  | 0.00022 |
| role:ics | 1 | 1434 | 5.47543 | 0.01942 | * | 0.00108 |
| pair:ics | 1 | 1434 | 31.80108 | 0.00000 | * | 0.00625 |
| rank:role:pair | 1 | 1434 | 12.32944 | 0.00046 | * | 0.00612 |
| rank:role:ics | 1 | 1434 | 0.07643 | 0.78223 |  | 0.00002 |
| rank:pair:ics | 1 | 1434 | 0.17391 | 0.67672 |  | 0.00003 |
| role:pair:ics | 1 | 1434 | 0.09814 | 0.75412 |  | 0.00002 |
| rank:role:pair:ics | 1 | 1434 | 6.36995 | 0.01171 | * | 0.00126 |

**Table S6.** ANOVA table for testing modulation depth to the grape-passenger distance. See Table S1 for abbreviations.

| Effect | DFn | DFd | F | p | p<.05 | ges |
| --- | --- | --- | --- | --- | --- | --- |
| rank | 1 | 1434 | 2.16373 | 0.14152 |  | 0.00114 |
| role | 1 | 1434 | 270.63450 | 0.00000 | * | 0.12468 |
| pair | 1 | 1434 | 4.19027 | 0.04084 | * | 0.00220 |
| ics | 1 | 1434 | 5.83122 | 0.01587 | * | 0.00100 |
| rank:role | 1 | 1434 | 2.34638 | 0.12579 |  | 0.00123 |
| rank:pair | 1 | 1434 | 0.52561 | 0.46858 |  | 0.00028 |
| role:pair | 1 | 1434 | 0.00523 | 0.94238 |  | 0.00000 |
| rank:ics | 1 | 1434 | 2.10089 | 0.14743 |  | 0.00036 |
| role:ics | 1 | 1434 | 26.48746 | 0.00000 | * | 0.00451 |
| pair:ics | 1 | 1434 | 0.36876 | 0.54378 |  | 0.00006 |
| rank:role:pair | 1 | 1434 | 0.01989 | 0.88786 |  | 0.00001 |
| rank:role:ics | 1 | 1434 | 12.14730 | 0.00051 | * | 0.00207 |
| rank:pair:ics | 1 | 1434 | 14.01678 | 0.00019 | * | 0.00239 |
| role:pair:ics | 1 | 1434 | 9.88632 | 0.00170 | * | 0.00169 |
| rank:role:pair:ics | 1 | 1434 | 0.26280 | 0.60828 |  | 0.00004 |

**Table S7.** ANOVA table for testing neuronal firing rates when the grape-passenger distance was less than 1m. See Table S1 for abbreviations.

| Effect | DFn | DFd | F | p | p<.05 | ges |
| --- | --- | --- | --- | --- | --- | --- |
| rank | 1 | 1434 | 54.31283 | 0.00000 | * | 0.02153 |
| role | 1 | 1434 | 4.52971 | 0.03348 | * | 0.00183 |
| pair | 1 | 1434 | 41.78852 | 0.00000 | * | 0.01665 |
| ics | 1 | 1434 | 610.93061 | 0.00000 | * | 0.15151 |
| rank:role | 1 | 1434 | 168.69844 | 0.00000 | * | 0.06396 |
| rank:pair | 1 | 1434 | 17.65991 | 0.00003 | * | 0.00710 |
| role:pair | 1 | 1434 | 0.14441 | 0.70399 |  | 0.00006 |
| rank:ics | 1 | 1434 | 35.72465 | 0.00000 | * | 0.01033 |
| role:ics | 1 | 1434 | 21.79602 | 0.00000 | * | 0.00633 |
| pair:ics | 1 | 1434 | 31.46990 | 0.00000 | * | 0.00911 |
| rank:role:pair | 1 | 1434 | 19.72990 | 0.00001 | * | 0.00793 |
| rank:role:ics | 1 | 1434 | 30.83873 | 0.00000 | * | 0.00893 |
| rank:pair:ics | 1 | 1434 | 0.67340 | 0.41200 |  | 0.00020 |
| role:pair:ics | 1 | 1434 | 10.70344 | 0.00109 | * | 0.00312 |
| rank:role:pair:ics | 1 | 1434 | 15.34405 | 0.00009 | * | 0.00446 |

**Table S8.** ANOVA table for testing flip-index for monkey pair C-K. See Table S1 for abbreviations.

| Effect | DFn | DFd | F | p | p<.05 | ges |
| --- | --- | --- | --- | --- | --- | --- |
| rank | 1 | 218 | 14.43229 | 0.00019 | * | 0.04417 |
| area | 1 | 218 | 0.01985 | 0.88809 |  | 0.00006 |
| role | 1 | 218 | 1.72969 | 0.18983 |  | 0.00551 |
| ics | 1 | 218 | 0.42082 | 0.51721 |  | 0.00058 |
| rank:area | 1 | 218 | 5.46597 | 0.02030 | * | 0.01720 |
| rank:role | 1 | 218 | 21.39642 | 0.00001 | * | 0.06412 |
| area:role | 1 | 218 | 1.99176 | 0.15958 |  | 0.00634 |
| rank:ics | 1 | 218 | 20.73875 | 0.00001 | * | 0.02792 |
| area:ics | 1 | 218 | 3.73783 | 0.05449 |  | 0.00515 |
| role:ics | 1 | 218 | 2.09994 | 0.14874 |  | 0.00290 |
| rank:area:role | 1 | 218 | 0.52791 | 0.46826 |  | 0.00169 |
| rank:area:ics | 1 | 218 | 5.91655 | 0.01581 | * | 0.00813 |
| rank:role:ics | 1 | 218 | 23.94291 | 0.00000 | * | 0.03210 |
| area:role:ics | 1 | 218 | 0.30609 | 0.58066 |  | 0.00042 |
| rank:area:role:ics | 1 | 218 | 0.05243 | 0.81910 |  | 0.00007 |

**Table S9.** Modulation depth for wheelchair velocity, acceleration, and position as the function of multiple parameters: cortical area (M1 or PMd), monkey pair, monkey role (passenger or observer), and the presence or absence of ICS.

| Pair | Role | Variate | Area | ICS | % modulated | depth | 95% conf. |
| --- | --- | --- | --- | --- | --- | --- | --- |
| C-K | passenger | velocity | monkeyC | + | 53.5% (115/215) | 0.022 | 0.019 - 0.024 |
|  |  |  |  | - | 51.2% (110/215) | 0.03 | 0.028 - 0.034 |
|  |  |  |  | all | 44.7% (96/215) | 0.027 | 0.025 - 0.030 |
|  |  |  | monkeyC_M1 | + | 47.8% (64/134) | 0.018 | 0.015 - 0.021 |
|  |  |  |  | - | 46.3% (62/134) | 0.027 | 0.023 - 0.031 |
|  |  |  |  | all | 37.3% (50/134) | 0.024 | 0.021 - 0.027 |
|  |  |  | monkeyC_PMd | + | 63.0% (51/81) | 0.028 | 0.024 - 0.033 |
|  |  |  |  | - | 59.3% (48/81) | 0.036 | 0.031 - 0.042 |
|  |  |  |  | all | 56.8% (46/81) | 0.033 | 0.029 - 0.038 |
|  |  |  | monkeyK | + | 52.1% (98/188) | 0.034 | 0.028 - 0.040 |
|  |  |  |  | - | 31.9% (60/188) | 0.028 | 0.024 - 0.033 |
|  |  |  |  | all | 34.0% (64/188) | 0.03 | 0.025 - 0.034 |
|  |  |  | monkeyK_M1 | + | 48.5% (63/130) | 0.029 | 0.023 - 0.035 |
|  |  |  |  | - | 29.2% (38/130) | 0.024 | 0.020 - 0.028 |
|  |  |  |  | all | 30.8% (40/130) | 0.025 | 0.021 - 0.030 |
|  |  |  | monkeyK_PMd | + | 60.3% (35/58) | 0.044 | 0.033 - 0.057 |
|  |  |  |  | - | 37.9% (22/58) | 0.039 | 0.029 - 0.049 |
|  |  |  |  | all | 41.4% (24/58) | 0.04 | 0.031 - 0.051 |
|  |  | acceleration | monkeyC | + | 37.2% (80/215) | 0.015 | 0.013 - 0.017 |
|  |  |  |  | - | 32.1% (69/215) | 0.021 | 0.019 - 0.024 |
|  |  |  |  | all | 22.3% (48/215) | 0.019 | 0.017 - 0.021 |
|  |  |  | monkeyC_M1 | + | 29.9% (40/134) | 0.012 | 0.009 - 0.015 |
|  |  |  |  | - | 29.9% (40/134) | 0.02 | 0.016 - 0.023 |
|  |  |  |  | all | 19.4% (26/134) | 0.017 | 0.015 - 0.020 |
|  |  |  | monkeyC_PMd | + | 49.4% (40/81) | 0.018 | 0.015 - 0.022 |
|  |  |  |  | - | 35.8% (29/81) | 0.024 | 0.020 - 0.029 |
|  |  |  |  | all | 27.2% (22/81) | 0.022 | 0.018 - 0.026 |
|  |  |  | monkeyK | + | 36.2% (68/188) | 0.02 | 0.016 - 0.025 |
|  |  |  |  | - | 14.4% (27/188) | 0.02 | 0.017 - 0.024 |
|  |  |  |  | all | 13.3% (25/188) | 0.02 | 0.017 - 0.024 |
|  |  |  | monkeyK_M1 | + | 33.8% (44/130) | 0.016 | 0.012 - 0.021 |
|  |  |  |  | - | 11.5% (15/130) | 0.016 | 0.013 - 0.020 |
|  |  |  |  | all | 10.8% (14/130) | 0.016 | 0.013 - 0.019 |
|  |  |  | monkeyK_PMd | + | 41.4% (24/58) | 0.029 | 0.021 - 0.038 |
|  |  |  |  | - | 20.7% (12/58) | 0.029 | 0.021 - 0.038 |
|  |  |  |  | all | 19.0% (11/58) | 0.029 | 0.020 - 0.038 |
|  |  | position | monkeyC | + | 80.9% (174/215) | 0.047 | 0.041 - 0.053 |
|  |  |  |  | - | 71.2% (153/215) | 0.045 | 0.041 - 0.050 |
|  |  |  |  | all | 67.4% (145/215) | 0.045 | 0.040 - 0.051 |
|  |  |  | monkeyC_M1 | + | 75.4% (101/134) | 0.046 | 0.039 - 0.056 |
|  |  |  |  | - | 66.4% (89/134) | 0.044 | 0.037 - 0.051 |
|  |  |  |  | all | 61.9% (83/134) | 0.044 | 0.038 - 0.052 |
|  |  |  | monkeyC_PMd | + | 90.1% (73/81) | 0.048 | 0.041 - 0.055 |
|  |  |  |  | - | 79.0% (64/81) | 0.048 | 0.042 - 0.055 |
|  |  |  |  | all | 76.5% (62/81) | 0.047 | 0.041 - 0.054 |
|  |  |  | monkeyK | + | 57.4% (108/188) | 0.048 | 0.039 - 0.057 |
|  |  |  |  | - | 36.2% (68/188) | 0.041 | 0.033 - 0.048 |
|  |  |  |  | all | 36.2% (68/188) | 0.041 | 0.034 - 0.049 |
|  |  |  | monkeyK_M1 | + | 53.1% (69/130) | 0.043 | 0.033 - 0.054 |
|  |  |  |  | - | 31.5% (41/130) | 0.036 | 0.027 - 0.045 |
|  |  |  |  | all | 31.5% (41/130) | 0.036 | 0.027 - 0.046 |
|  |  |  | monkeyK_PMd | + | 67.2% (39/58) | 0.059 | 0.043 - 0.076 |
|  |  |  |  | - | 46.6% (27/58) | 0.051 | 0.038 - 0.064 |
|  |  |  |  | all | 46.6% (27/58) | 0.052 | 0.038 - 0.068 |
|  | observer | velocity | monkeyC | + | 18.3% (51/279) | 0.013 | 0.011 - 0.014 |
|  |  |  |  | - | 0.0% (0/279) | 0.003 | 0.002 - 0.003 |
|  |  |  |  | all | 0.0% (0/279) | 0.003 | 0.003 - 0.004 |
|  |  |  | monkeyC_M1 | + | 18.7% (32/171) | 0.013 | 0.011 - 0.015 |
|  |  |  |  | - | 0.0% (0/171) | 0.003 | 0.002 - 0.004 |
|  |  |  |  | all | 0.0% (0/171) | 0.003 | 0.002 - 0.004 |
|  |  |  | monkeyC_PMd | + | 17.6% (19/108) | 0.012 | 0.009 - 0.015 |
|  |  |  |  | - | 0.0% (0/108) | 0.003 | 0.002 - 0.004 |
|  |  |  |  | all | 0.0% (0/108) | 0.003 | 0.002 - 0.004 |
|  |  |  | monkeyK | + | 21.4% (30/140) | 0.009 | 0.006 - 0.011 |
|  |  |  |  | - | 2.1% (3/140) | 0.005 | 0.004 - 0.007 |
|  |  |  |  | all | 2.1% (3/140) | 0.005 | 0.004 - 0.007 |
|  |  |  | monkeyK_M1 | + | 20.8% (20/96) | 0.009 | 0.006 - 0.011 |
|  |  |  |  | - | 2.1% (2/96) | 0.006 | 0.004 - 0.008 |
|  |  |  |  | all | 2.1% (2/96) | 0.006 | 0.004 - 0.007 |
|  |  |  | monkeyK_PMd | + | 22.7% (10/44) | 0.009 | 0.004 - 0.014 |
|  |  |  |  | - | 2.3% (1/44) | 0.005 | 0.002 - 0.007 |
|  |  |  |  | all | 2.3% (1/44) | 0.004 | 0.002 - 0.007 |
|  |  | acceleration | monkeyC | + | 0.0% (0/279) | 0 | -0.003 |
|  |  |  |  | - | 0.0% (0/279) | -0.001 | -0.001 |
|  |  |  |  | all | 0.0% (0/279) | 0 | 0 |
|  |  |  | monkeyC_M1 | + | 0.0% (0/171) | 0.001 | -0.005 |
|  |  |  |  | - | 0.0% (0/171) | 0 | -0.001 |
|  |  |  |  | all | 0.0% (0/171) | 0 | 0 |
|  |  |  | monkeyC_PMd | + | 0.0% (0/108) | -0.002 | -0.004 |
|  |  |  |  | - | 0.0% (0/108) | -0.001 | -0.001 |
|  |  |  |  | all | 0.0% (0/108) | 0 | -0.001 |
|  |  |  | monkeyK | + | 0.7% (1/140) | -0.001 | -0.003 |
|  |  |  |  | - | 0.0% (0/140) | 0 | -0.001 |
|  |  |  |  | all | 0.0% (0/140) | 0 | -0.001 |
|  |  |  | monkeyK_M1 | + | 0.0% (0/96) | 0 | -0.004 |
|  |  |  |  | - | 0.0% (0/96) | 0 | -0.001 |
|  |  |  |  | all | 0.0% (0/96) | 0 | -0.001 |
|  |  |  | monkeyK_PMd | + | 2.3% (1/44) | -0.002 | -0.006 |
|  |  |  |  | - | 0.0% (0/44) | 0 | -0.002 |
|  |  |  |  | all | 0.0% (0/44) | 0 | -0.002 |
|  |  | position | monkeyC | + | 57.3% (160/279) | 0.026 | 0.023 - 0.028 |
|  |  |  |  | - | 0.0% (0/279) | 0.008 | 0.007 - 0.009 |
|  |  |  |  | all | 0.0% (0/279) | 0.008 | 0.007 - 0.009 |
|  |  |  | monkeyC_M1 | + | 52.6% (90/171) | 0.022 | 0.019 - 0.025 |
|  |  |  |  | - | 0.0% (0/171) | 0.007 | 0.006 - 0.009 |
|  |  |  |  | all | 0.0% (0/171) | 0.007 | 0.006 - 0.008 |
|  |  |  | monkeyC_PMd | + | 64.8% (70/108) | 0.031 | 0.027 - 0.035 |
|  |  |  |  | - | 0.0% (0/108) | 0.009 | 0.007 - 0.010 |
|  |  |  |  | all | 0.0% (0/108) | 0.01 | 0.008 - 0.011 |
|  |  |  | monkeyK | + | 64.3% (90/140) | 0.042 | 0.035 - 0.049 |
|  |  |  |  | - | 56.4% (79/140) | 0.041 | 0.034 - 0.048 |
|  |  |  |  | all | 53.6% (75/140) | 0.043 | 0.036 - 0.051 |
|  |  |  | monkeyK_M1 | + | 61.5% (59/96) | 0.041 | 0.033 - 0.051 |
|  |  |  |  | - | 56.2% (54/96) | 0.042 | 0.034 - 0.052 |
|  |  |  |  | all | 53.1% (51/96) | 0.044 | 0.034 - 0.055 |
|  |  |  | monkeyK_PMd | + | 70.5% (31/44) | 0.042 | 0.032 - 0.053 |
|  |  |  |  | - | 56.8% (25/44) | 0.038 | 0.028 - 0.048 |
|  |  |  |  | all | 54.5% (24/44) | 0.042 | 0.031 - 0.054 |
| C-J | passenger | velocity | monkeyC | + | 57.7% (101/175) | 0.031 | 0.027 - 0.036 |
|  |  |  |  | - | 42.3% (74/175) | 0.028 | 0.023 - 0.032 |
|  |  |  |  | all | 40.6% (71/175) | 0.028 | 0.024 - 0.033 |
|  |  |  | monkeyC_M1 | + | 72.8% (75/103) | 0.04 | 0.034 - 0.045 |
|  |  |  |  | - | 52.4% (54/103) | 0.032 | 0.027 - 0.038 |
|  |  |  |  | all | 52.4% (54/103) | 0.035 | 0.030 - 0.040 |
|  |  |  | monkeyC_PMd | + | 36.1% (26/72) | 0.019 | 0.013 - 0.025 |
|  |  |  |  | - | 27.8% (20/72) | 0.02 | 0.014 - 0.028 |
|  |  |  |  | all | 23.6% (17/72) | 0.019 | 0.013 - 0.025 |
|  |  |  | monkeyJ | + | 68.4% (91/133) | 0.044 | 0.037 - 0.050 |
|  |  |  |  | - | 56.4% (75/133) | 0.039 | 0.034 - 0.045 |
|  |  |  |  | all | 54.9% (73/133) | 0.039 | 0.034 - 0.045 |
|  |  |  | monkeyJ_M1 | + | 68.4% (91/133) | 0.044 | 0.038 - 0.050 |
|  |  |  |  | - | 56.4% (75/133) | 0.039 | 0.034 - 0.044 |
|  |  |  |  | all | 54.9% (73/133) | 0.039 | 0.034 - 0.044 |
|  |  | acceleration | monkeyC | + | 43.4% (76/175) | 0.021 | 0.017 - 0.025 |
|  |  |  |  | - | 20.0% (35/175) | 0.016 | 0.013 - 0.019 |
|  |  |  |  | all | 19.4% (34/175) | 0.018 | 0.014 - 0.021 |
|  |  |  | monkeyC_M1 | + | 53.4% (55/103) | 0.028 | 0.023 - 0.034 |
|  |  |  |  | - | 26.2% (27/103) | 0.02 | 0.016 - 0.025 |
|  |  |  |  | all | 24.3% (25/103) | 0.023 | 0.018 - 0.028 |
|  |  |  | monkeyC_PMd | + | 29.2% (21/72) | 0.01 | 0.006 - 0.015 |
|  |  |  |  | - | 11.1% (8/72) | 0.01 | 0.006 - 0.014 |
|  |  |  |  | all | 12.5% (9/72) | 0.01 | 0.007 - 0.013 |
|  |  |  | monkeyJ | + | 32.3% (43/133) | 0.016 | 0.013 - 0.020 |
|  |  |  |  | - | 18.0% (24/133) | 0.02 | 0.017 - 0.023 |
|  |  |  |  | all | 7.5% (10/133) | 0.018 | 0.015 - 0.021 |
|  |  |  | monkeyJ_M1 | + | 32.3% (43/133) | 0.016 | 0.013 - 0.020 |
|  |  |  |  | - | 18.0% (24/133) | 0.02 | 0.017 - 0.023 |
|  |  |  |  | all | 7.5% (10/133) | 0.018 | 0.016 - 0.022 |
|  |  | position | monkeyC | + | 74.3% (130/175) | 0.051 | 0.044 - 0.059 |
|  |  |  |  | - | 68.6% (120/175) | 0.055 | 0.047 - 0.062 |
|  |  |  |  | all | 63.4% (111/175) | 0.05 | 0.043 - 0.056 |
|  |  |  | monkeyC_M1 | + | 89.3% (92/103) | 0.063 | 0.054 - 0.071 |
|  |  |  |  | - | 77.7% (80/103) | 0.062 | 0.052 - 0.070 |
|  |  |  |  | all | 72.8% (75/103) | 0.058 | 0.050 - 0.068 |
|  |  |  | monkeyC_PMd | + | 52.8% (38/72) | 0.035 | 0.025 - 0.046 |
|  |  |  |  | - | 55.6% (40/72) | 0.045 | 0.034 - 0.056 |
|  |  |  |  | all | 50.0% (36/72) | 0.037 | 0.028 - 0.047 |
|  |  |  | monkeyJ | + | 72.2% (96/133) | 0.052 | 0.045 - 0.059 |
|  |  |  |  | - | 56.4% (75/133) | 0.049 | 0.042 - 0.055 |
|  |  |  |  | all | 54.9% (73/133) | 0.047 | 0.040 - 0.053 |
|  |  |  | monkeyJ_M1 | + | 72.2% (96/133) | 0.052 | 0.044 - 0.059 |
|  |  |  |  | - | 56.4% (75/133) | 0.049 | 0.043 - 0.055 |
|  |  |  |  | all | 54.9% (73/133) | 0.047 | 0.041 - 0.053 |
|  | observer | velocity | monkeyC | + | 3.4% (6/178) | 0.003 | -0.006 |
|  |  |  |  | - | 0.0% (0/178) | 0.011 | 0.007 - 0.017 |
|  |  |  |  | all | 0.0% (0/178) | 0.005 | 0.003 - 0.007 |
|  |  |  | monkeyC_M1 | + | 2.8% (3/106) | 0.002 | -0.009 |
|  |  |  |  | - | 0.0% (0/106) | 0.015 | 0.008 - 0.025 |
|  |  |  |  | all | 0.0% (0/106) | 0.006 | 0.003 - 0.008 |
|  |  |  | monkeyC_PMd | + | 4.2% (3/72) | 0.006 | 0.002 - 0.009 |
|  |  |  |  | - | 0.0% (0/72) | 0.005 | 0.003 - 0.007 |
|  |  |  |  | all | 0.0% (0/72) | 0.004 | 0.003 - 0.005 |
|  |  |  | monkeyJ | + | 38.1% (51/134) | 0.017 | 0.015 - 0.020 |
|  |  |  |  | - | 10.4% (14/134) | 0.005 | -0.027 |
|  |  |  |  | all | 0.0% (0/134) | 0.013 | 0.011 - 0.015 |
|  |  |  | monkeyJ_M1 | + | 38.1% (51/134) | 0.018 | 0.015 - 0.020 |
|  |  |  |  | - | 10.4% (14/134) | 0.005 | -0.027 |
|  |  |  |  | all | 0.0% (0/134) | 0.013 | 0.011 - 0.015 |
|  |  | acceleration | monkeyC | + | 0.0% (0/178) | -0.002 | -0.006 |
|  |  |  |  | - | 0.0% (0/178) | 0.001 | -0.01 |
|  |  |  |  | all | 0.0% (0/178) | 0 | -0.003 |
|  |  |  | monkeyC_M1 | + | 0.0% (0/106) | -0.003 | -0.009 |
|  |  |  |  | - | 0.0% (0/106) | 0.003 | -0.017 |
|  |  |  |  | all | 0.0% (0/106) | -0.001 | -0.005 |
|  |  |  | monkeyC_PMd | + | 0.0% (0/72) | 0 | -0.006 |
|  |  |  |  | - | 0.0% (0/72) | -0.001 | -0.002 |
|  |  |  |  | all | 0.0% (0/72) | 0 | -0.001 |
|  |  |  | monkeyJ | + | 4.5% (6/134) | 0.004 | 0.002 - 0.006 |
|  |  |  |  | - | 0.0% (0/134) | -0.007 | -0.025 |
|  |  |  |  | all | 0.0% (0/134) | 0.001 | 0.000 - 0.002 |
|  |  |  | monkeyJ_M1 | + | 4.5% (6/134) | 0.004 | 0.002 - 0.006 |
|  |  |  |  | - | 0.0% (0/134) | -0.007 | -0.026 |
|  |  |  |  | all | 0.0% (0/134) | 0.001 | 0.000 - 0.002 |
|  |  | position | monkeyC | + | 59.0% (105/178) | 0.029 | 0.025 - 0.034 |
|  |  |  |  | - | 17.4% (31/178) | 0.021 | 0.016 - 0.027 |
|  |  |  |  | all | 14.0% (25/178) | 0.018 | 0.015 - 0.020 |
|  |  |  | monkeyC_M1 | + | 66.0% (70/106) | 0.034 | 0.027 - 0.040 |
|  |  |  |  | - | 22.6% (24/106) | 0.025 | 0.018 - 0.034 |
|  |  |  |  | all | 16.0% (17/106) | 0.019 | 0.016 - 0.023 |
|  |  |  | monkeyC_PMd | + | 48.6% (35/72) | 0.022 | 0.017 - 0.028 |
|  |  |  |  | - | 9.7% (7/72) | 0.015 | 0.011 - 0.019 |
|  |  |  |  | all | 11.1% (8/72) | 0.015 | 0.012 - 0.019 |
|  |  |  | monkeyJ | + | 72.4% (97/134) | 0.038 | 0.033 - 0.043 |
|  |  |  |  | - | 56.7% (76/134) | 0.023 | 0.005 - 0.034 |
|  |  |  |  | all | 56.0% (75/134) | 0.032 | 0.028 - 0.037 |
|  |  |  | monkeyJ_M1 | + | 72.4% (97/134) | 0.038 | 0.032 - 0.042 |
|  |  |  |  | - | 56.7% (76/134) | 0.024 | 0.005 - 0.034 |
|  |  |  |  | all | 56.0% (75/134) | 0.033 | 0.028 - 0.037 |

**Table S10.** Modulation depth to observer-passenger distance and grape-passenger distance of M1 and PMd units when a monkey was either the passenger or the observer, and either in or out of ICS. O-P distance, observer-passenger distance; G-O distance, grape-observer distance.

| Pair | Role | Variate | Area | ICS | % modulated | depth | 95% |
| --- | --- | --- | --- | --- | --- | --- | --- |
| C-K | passenger | O-P distance | monkeyC | + | 48.4% (104/215) | 0.023 | 0.018 - 0.029 |
|  |  |  |  | - | 42.3% (91/215) | 0.028 | 0.024 - 0.033 |
|  |  |  |  | all | 31.2% (67/215) | 0.025 | 0.020 - 0.030 |
|  |  |  | monkeyC_M1 | + | 50.0% (67/134) | 0.026 | 0.018 - 0.036 |
|  |  |  |  | - | 40.3% (54/134) | 0.028 | 0.022 - 0.035 |
|  |  |  |  | all | 29.9% (40/134) | 0.026 | 0.019 - 0.033 |
|  |  |  | monkeyC_PMd | + | 45.7% (37/81) | 0.019 | 0.015 - 0.023 |
|  |  |  |  | - | 45.7% (37/81) | 0.028 | 0.023 - 0.032 |
|  |  |  |  | all | 33.3% (27/81) | 0.023 | 0.019 - 0.028 |
|  |  |  | monkeyK | + | 39.4% (74/188) | 0.027 | 0.022 - 0.032 |
|  |  |  |  | - | 11.2% (21/188) | 0.015 | 0.012 - 0.018 |
|  |  |  |  | all | 12.2% (23/188) | 0.015 | 0.013 - 0.019 |
|  |  |  | monkeyK_M1 | + | 36.2% (47/130) | 0.024 | 0.018 - 0.030 |
|  |  |  |  | - | 11.5% (15/130) | 0.014 | 0.011 - 0.018 |
|  |  |  |  | all | 11.5% (15/130) | 0.015 | 0.011 - 0.018 |
|  |  |  | monkeyK_PMd | + | 46.6% (27/58) | 0.032 | 0.023 - 0.041 |
|  |  |  |  | - | 10.3% (6/58) | 0.015 | 0.011 - 0.021 |
|  |  |  |  | all | 13.8% (8/58) | 0.017 | 0.012 - 0.022 |
|  |  | G-O distance | monkeyC | + | 80.5% (173/215) | 0.04 | 0.036 - 0.043 |
|  |  |  |  | - | 67.0% (144/215) | 0.041 | 0.037 - 0.045 |
|  |  |  |  | all | 62.8% (135/215) | 0.04 | 0.036 - 0.044 |
|  |  |  | monkeyC_M1 | + | 75.4% (101/134) | 0.036 | 0.031 - 0.040 |
|  |  |  |  | - | 60.4% (81/134) | 0.037 | 0.032 - 0.042 |
|  |  |  |  | all | 55.2% (74/134) | 0.036 | 0.032 - 0.041 |
|  |  |  | monkeyC_PMd | + | 88.9% (72/81) | 0.047 | 0.041 - 0.053 |
|  |  |  |  | - | 77.8% (63/81) | 0.047 | 0.041 - 0.054 |
|  |  |  |  | all | 75.3% (61/81) | 0.047 | 0.040 - 0.053 |
|  |  |  | monkeyK | + | 53.7% (101/188) | 0.046 | 0.038 - 0.055 |
|  |  |  |  | - | 38.8% (73/188) | 0.043 | 0.036 - 0.052 |
|  |  |  |  | all | 37.2% (70/188) | 0.044 | 0.036 - 0.052 |
|  |  |  | monkeyK_M1 | + | 50.0% (65/130) | 0.041 | 0.032 - 0.051 |
|  |  |  |  | - | 33.8% (44/130) | 0.038 | 0.028 - 0.048 |
|  |  |  |  | all | 31.5% (41/130) | 0.038 | 0.029 - 0.048 |
|  |  |  | monkeyK_PMd | + | 62.1% (36/58) | 0.059 | 0.044 - 0.075 |
|  |  |  |  | - | 50.0% (29/58) | 0.056 | 0.042 - 0.072 |
|  |  |  |  | all | 50.0% (29/58) | 0.057 | 0.041 - 0.074 |
|  | observer | O-P distance | monkeyC | + | 25.1% (70/279) | 0.014 | 0.012 - 0.016 |
|  |  |  |  | - | 0.0% (0/279) | 0.006 | 0.005 - 0.008 |
|  |  |  |  | all | 0.0% (0/279) | 0.005 | 0.004 - 0.006 |
|  |  |  | monkeyC_M1 | + | 22.2% (38/171) | 0.013 | 0.010 - 0.016 |
|  |  |  |  | - | 0.0% (0/171) | 0.006 | 0.005 - 0.007 |
|  |  |  |  | all | 0.0% (0/171) | 0.005 | 0.004 - 0.006 |
|  |  |  | monkeyC_PMd | + | 29.6% (32/108) | 0.016 | 0.013 - 0.019 |
|  |  |  |  | - | 0.0% (0/108) | 0.007 | 0.006 - 0.009 |
|  |  |  |  | all | 0.0% (0/108) | 0.006 | 0.005 - 0.007 |
|  |  |  | monkeyK | + | 62.9% (88/140) | 0.043 | 0.036 - 0.050 |
|  |  |  |  | - | 51.4% (72/140) | 0.039 | 0.032 - 0.046 |
|  |  |  |  | all | 52.1% (73/140) | 0.042 | 0.035 - 0.050 |
|  |  |  | monkeyK_M1 | + | 60.4% (58/96) | 0.042 | 0.034 - 0.051 |
|  |  |  |  | - | 52.1% (50/96) | 0.04 | 0.031 - 0.050 |
|  |  |  |  | all | 50.0% (48/96) | 0.043 | 0.033 - 0.054 |
|  |  |  | monkeyK_PMd | + | 68.2% (30/44) | 0.044 | 0.033 - 0.056 |
|  |  |  |  | - | 50.0% (22/44) | 0.036 | 0.026 - 0.047 |
|  |  |  |  | all | 56.8% (25/44) | 0.041 | 0.030 - 0.053 |
|  |  | G-P distance | monkeyC | + | 38.4% (107/279) | 0.019 | 0.016 - 0.021 |
|  |  |  |  | - | 0.0% (0/279) | 0.006 | 0.005 - 0.007 |
|  |  |  |  | all | 0.0% (0/279) | 0.007 | 0.006 - 0.008 |
|  |  |  | monkeyC_M1 | + | 34.5% (59/171) | 0.017 | 0.014 - 0.020 |
|  |  |  |  | - | 0.0% (0/171) | 0.006 | 0.005 - 0.007 |
|  |  |  |  | all | 0.0% (0/171) | 0.006 | 0.005 - 0.007 |
|  |  |  | monkeyC_PMd | + | 44.4% (48/108) | 0.021 | 0.017 - 0.026 |
|  |  |  |  | - | 0.0% (0/108) | 0.007 | 0.006 - 0.009 |
|  |  |  |  | all | 0.0% (0/108) | 0.008 | 0.006 - 0.010 |
|  |  |  | monkeyK | + | 18.6% (26/140) | 0.01 | 0.007 - 0.014 |
|  |  |  |  | - | 8.6% (12/140) | 0.011 | 0.009 - 0.014 |
|  |  |  |  | all | 7.1% (10/140) | 0.01 | 0.008 - 0.013 |
|  |  |  | monkeyK_M1 | + | 17.7% (17/96) | 0.009 | 0.005 - 0.014 |
|  |  |  |  | - | 8.3% (8/96) | 0.012 | 0.008 - 0.016 |
|  |  |  |  | all | 6.2% (6/96) | 0.01 | 0.007 - 0.014 |
|  |  |  | monkeyK_PMd | + | 20.5% (9/44) | 0.011 | 0.007 - 0.016 |
|  |  |  |  | - | 9.1% (4/44) | 0.01 | 0.006 - 0.014 |
|  |  |  |  | all | 9.1% (4/44) | 0.01 | 0.007 - 0.015 |
| C-J | passenger | O-P distance | monkeyC | + | 56.0% (98/175) | 0.028 | 0.024 - 0.033 |
|  |  |  |  | - | 46.9% (82/175) | 0.029 | 0.025 - 0.033 |
|  |  |  |  | all | 36.6% (64/175) | 0.026 | 0.022 - 0.031 |
|  |  |  | monkeyC_M1 | + | 63.1% (65/103) | 0.034 | 0.028 - 0.040 |
|  |  |  |  | - | 48.5% (50/103) | 0.034 | 0.028 - 0.040 |
|  |  |  |  | all | 41.7% (43/103) | 0.031 | 0.025 - 0.037 |
|  |  |  | monkeyC_PMd | + | 45.8% (33/72) | 0.021 | 0.015 - 0.028 |
|  |  |  |  | - | 44.4% (32/72) | 0.022 | 0.016 - 0.028 |
|  |  |  |  | all | 29.2% (21/72) | 0.02 | 0.015 - 0.025 |
|  |  |  | monkeyJ | + | 59.4% (79/133) | 0.038 | 0.032 - 0.044 |
|  |  |  |  | - | 45.1% (60/133) | 0.036 | 0.031 - 0.041 |
|  |  |  |  | all | 44.4% (59/133) | 0.035 | 0.030 - 0.040 |
|  |  |  | monkeyJ_M1 | + | 59.4% (79/133) | 0.038 | 0.032 - 0.044 |
|  |  |  |  | - | 45.1% (60/133) | 0.035 | 0.030 - 0.041 |
|  |  |  |  | all | 44.4% (59/133) | 0.035 | 0.029 - 0.040 |
|  |  | G-P distance | monkeyC | + | 53.7% (94/175) | 0.035 | 0.029 - 0.041 |
|  |  |  |  | - | 64.0% (112/175) | 0.051 | 0.044 - 0.059 |
|  |  |  |  | all | 53.1% (93/175) | 0.043 | 0.037 - 0.050 |
|  |  |  | monkeyC_M1 | + | 62.1% (64/103) | 0.044 | 0.035 - 0.053 |
|  |  |  |  | - | 69.9% (72/103) | 0.059 | 0.049 - 0.068 |
|  |  |  |  | all | 59.2% (61/103) | 0.051 | 0.043 - 0.061 |
|  |  |  | monkeyC_PMd | + | 41.7% (30/72) | 0.023 | 0.016 - 0.031 |
|  |  |  |  | - | 55.6% (40/72) | 0.04 | 0.031 - 0.051 |
|  |  |  |  | all | 44.4% (32/72) | 0.032 | 0.024 - 0.041 |
|  |  |  | monkeyJ | + | 72.9% (97/133) | 0.051 | 0.044 - 0.058 |
|  |  |  |  | - | 60.9% (81/133) | 0.049 | 0.043 - 0.056 |
|  |  |  |  | all | 54.9% (73/133) | 0.048 | 0.042 - 0.054 |
|  |  |  | monkeyJ_M1 | + | 72.9% (97/133) | 0.051 | 0.043 - 0.058 |
|  |  |  |  | - | 60.9% (81/133) | 0.049 | 0.042 - 0.055 |
|  |  |  |  | all | 54.9% (73/133) | 0.048 | 0.042 - 0.055 |
|  | observer | O-P distance | monkeyC | + | 53.9% (96/178) | 0.028 | 0.023 - 0.033 |
|  |  |  |  | - | 23.0% (41/178) | 0.024 | 0.020 - 0.031 |
|  |  |  |  | all | 22.5% (40/178) | 0.023 | 0.019 - 0.026 |
|  |  |  | monkeyC_M1 | + | 61.3% (65/106) | 0.032 | 0.024 - 0.039 |
|  |  |  |  | - | 28.3% (30/106) | 0.029 | 0.021 - 0.039 |
|  |  |  |  | all | 28.3% (30/106) | 0.025 | 0.021 - 0.030 |
|  |  |  | monkeyC_PMd | + | 43.1% (31/72) | 0.022 | 0.016 - 0.029 |
|  |  |  |  | - | 15.3% (11/72) | 0.018 | 0.013 - 0.023 |
|  |  |  |  | all | 13.9% (10/72) | 0.018 | 0.013 - 0.024 |
|  |  |  | monkeyJ | + | 42.5% (57/134) | 0.019 | 0.016 - 0.022 |
|  |  |  |  | - | 27.6% (37/134) | 0.013 | -0.031 |
|  |  |  |  | all | 20.1% (27/134) | 0.019 | 0.016 - 0.023 |
|  |  |  | monkeyJ_M1 | + | 42.5% (57/134) | 0.019 | 0.015 - 0.022 |
|  |  |  |  | - | 27.6% (37/134) | 0.013 | -0.03 |
|  |  |  |  | all | 20.1% (27/134) | 0.019 | 0.016 - 0.023 |
|  |  | G-P distance | monkeyC | + | 35.4% (63/178) | 0.019 | 0.014 - 0.023 |
|  |  |  |  | - | 0.0% (0/178) | 0.01 | 0.006 - 0.016 |
|  |  |  |  | all | 0.0% (0/178) | 0.008 | 0.006 - 0.009 |
|  |  |  | monkeyC_M1 | + | 46.2% (49/106) | 0.025 | 0.017 - 0.031 |
|  |  |  |  | - | 0.0% (0/106) | 0.014 | 0.007 - 0.023 |
|  |  |  |  | all | 0.0% (0/106) | 0.009 | 0.007 - 0.011 |
|  |  |  | monkeyC_PMd | + | 19.4% (14/72) | 0.01 | 0.006 - 0.015 |
|  |  |  |  | - | 0.0% (0/72) | 0.005 | 0.003 - 0.007 |
|  |  |  |  | all | 0.0% (0/72) | 0.006 | 0.004 - 0.008 |
|  |  |  | monkeyJ | + | 50.0% (67/134) | 0.023 | 0.019 - 0.027 |
|  |  |  |  | - | 26.9% (36/134) | 0.01 | -0.028 |
|  |  |  |  | all | 14.2% (19/134) | 0.018 | 0.015 - 0.021 |
|  |  |  | monkeyJ_M1 | + | 50.0% (67/134) | 0.023 | 0.019 - 0.027 |
|  |  |  |  | - | 26.9% (36/134) | 0.009 | -0.028 |
|  |  |  |  | all | 14.2% (19/134) | 0.018 | 0.015 - 0.021 |
